# Macrophage Paired Immunoglobulin-Like Receptor B Deficiency Promotes Peripheral Atherosclerosis in Apolipoprotein E–Deficient Mice

**DOI:** 10.3389/fcell.2021.783954

**Published:** 2022-03-07

**Authors:** Wenhua Su, Liwen Liang, Liang Zhou, Yu Cao, Xiuli Zhou, Shiqi Liu, Qian Wang, Hong Zhang

**Affiliations:** ^1^ Department of Cardiology, First People’s Hospital of Yunnan Province, Kunming, China; ^2^ Faculty of Life Science and Biotechnology, Kunming University of Science and Technology, Kunming, China; ^3^ Department of Cardiovascular Surgery, First People’s Hospital of Yunnan Province, Kunming, China

**Keywords:** atherosclerosis, PAD, LILRB2, PirB, apolipoprotein

## Abstract

**Background:** Peripheral atherosclerotic disease (PAD) is the narrowing or blockage of arteries that supply blood to the lower limbs. Given its complex nature, bioinformatics can help identify crucial genes involved in the progression of peripheral atherosclerosis.

**Materials and Methods:** Raw human gene expression data for 462 PAD arterial plaque and 23 normal arterial samples were obtained from the GEO database. The data was analyzed using an integrated, multi-layer approach involving differentially-expressed gene analysis, KEGG pathway analysis, GO term enrichment analysis, weighted gene correlation network analysis, and protein-protein interaction analysis. The monocyte/macrophage-expressed leukocyte immunoglobulin-like receptor B2 (LILRB2) was strongly associated with the human PAD phenotype. To explore the role of the murine LILRB2 homologue PirB *in vivo*, we created a myeloid-specific *PirB*-knockout *Apoe* ^−/−^ murine model of PAD (*PirB* ^MΦKO^) to analyze femoral atherosclerotic burden, plaque features of vulnerability, and monocyte recruitment to femoral atherosclerotic lesions. The phenotypes of *PirB* ^MΦKO^ macrophages under various stimuli were also investigated *in vitro*.

**Results:** *PirB* ^MΦKO^ mice displayed increased femoral atherogenesis, a more vulnerable plaque phenotype, and enhanced monocyte recruitment into lesions. *PirB* ^MΦKO^ macrophages showed enhanced pro-inflammatory responses and a shift toward M1 over M2 polarization under interferon-γ and oxidized LDL exposure. *PirB* ^MΦKO^ macrophages also displayed enhanced efferocytosis and reduced lipid efflux under lipid exposure.

**Conclusion:** Macrophage PirB reduces peripheral atherosclerotic burden, stabilizes peripheral plaque composition, and suppresses macrophage accumulation in peripheral lesions. Macrophage PirB inhibits pro-inflammatory activation, inhibits efferocytosis, and promotes lipid efflux, characteristics critical to suppressing peripheral atherogenesis.

## Background

The narrowing or blockage of arteries that supply blood to the lower limbs is known as peripheral atherosclerotic disease (PAD). The principal cause of PAD is the atherosclerotic occlusion of arteries supplying the affected limbs. Although the disease is mostly asymptomatic, a commonplace clinical presentation is intermittent claudication (i.e., pain on walking). More severe clinical manifestations include critical limb ischemia (CLI), which presents as pain even during rest as well as tissue loss due to ulceration or gangrene (Morley et al., 2018). PAD is estimated to affect about 13% in adults of the Western population aged 50 or above (Morley et al., 2018). Mortality due to cardiovascular disease is seen in 10–15% of patients with intermittent claudication within 5 years of diagnosis (Norgren et al., 2007). Based on this evidence, it is important to identify the pathophysiological mechanism(s) underlying PAD progression, which can provide guidance towards more effective management of PAD patients.

However, the molecular pathophysiology underlying PAD is complicated, as there are a number of pathways, proteins, and cell types involved in PAD progression (Scholz et al., 2002; Coats and Wadsworth, 2005; Kuang et al., 2008). The primary cells implicated in the development of PAD include macrophages, vascular endothelial cells (ECs), resident stem cells, platelets, vascular smooth muscle cells (SMCs), fibroblasts, and pericytes (Scholz et al., 2002; Coats and Wadsworth, 2005; Kuang et al., 2008). In an otherwise healthy individual, tissue damage due to progressive limb ischemia progresses along a continuum. Initially, the body attempts to homeostatically restore blood supply to the affected limb(s) by angiogenic and arteriogenic pathways. To further resolve limb ischemia and tissue damage, inflammatory, vascular remodeling, and apoptotic pathways are activated. However, in patients diagnosed with CLI, such compensatory mechanisms are inefficient to restore sufficient blood flow. Due to this, there is continued inadequacy in perfusion coupled with enhanced chronic inflammation, EC dysfunction, and oxidative stress. Gangrene can then present as a consequence of muscle fiber damage due to persistently high levels of oxidative stress (Hickman et al., 1994; Bhat et al., 1999; Pipinos et al., 2008b; a;Koutakis et al., 2015).

Given the complex nature of the molecular pathways at play in PAD, bioinformatics analysis of arterial gene expression datasets from PAD patients and healthy individuals can help in identifying crucial genes involved in the progression of peripheral atherosclerosis. To this end, we obtained raw gene expression data for 462 PAD arterial plaque samples and 23 normal arterial samples from the GEO database. We analyzed the data using an integrated, multi-layer approach involving differentially-expressed gene (DEG) analysis, Kyoto Encyclopedia of Genes and Genomes (KEGG) pathway analysis, Gene Ontology (GO) term enrichment analysis, weighted Gene Correlation Network Analysis (WGCNA), and protein-protein interaction (PPI) network analysis (Bindea et al., 2013; Szklarczyk et al., 2016). Based on this integrated approach, we discovered the inhibitory monocyte/macrophage-expressed receptor—leukocyte immunoglobulin-like receptor B2 (LILRB2, LIR-2, ILT-4; ENSG00000131042, human chromosomal region 19q13.4)—to be strongly associated with the PAD phenotype. We then investigated the role of the murine analogue of human LILRB2, paired immunoglobulin-like receptor B (PirB, Lilrb3, Lir-3, Gp91; ENSMUSG00000058818, mouse chr7: 3,711,409-3,720,391(-)), in a myeloid-specific *PirB*-null *Apoe* ^−/−^ murine model of PAD. The application of our integrated approach can help provide much-needed guidance into the molecular mechanisms underlying PAD and other forms of atherosclerotic disease.

## Methods

The experimental methods are fully detailed in the Supplementary Material.

## Results

### DEGs in PAD and Their Functional Analysis

Application of DEG cut-off thresholds (log|fold-change (FC)| ≥ 1.5 and adjusted *p* < 0.05) following data processing of 13,467 common genes (Supplementary Figure S1; Supplementary File S2A) yielded a total of 680 DEGs, with 545 genes upregulated and 135 genes downregulated in PAD samples as compared with normal controls (Supplementary File S2B). Functional enrichment analysis of the 680 DEGs using gene ontology (GO, sub-ontologies: BP, CC, MF) and KEGG identified 21 enriched GO terms and 14 enriched KEGG pathways (Supplementary Figure S2; Supplementary File S3). The GO terms that were most significantly enriched included positive regulation of T-cell proliferation (GO_BP, *p* = 2.16E-15), positive regulation of phagocytosis (GO_BP, *p* = 4.89E-12), and tertiary granule membrane (GO_CC, *p* = 2.28E-11). The top-ranking KEGG pathways were hematopoietic cell lineage (*p* = 6.15E-18), rheumatoid arthritis (*p* = 1.68E-12), and *Staphylococcus aureus* infection (*p* = 5.37E-12).

### WGCNA Analysis

For WGCNA analysis, we took the variance-filtered genes (≥50%) from the 485 samples that were batch-corrected using the ComBat algorithm. A soft-threshold power of 5 was chosen using the function pickSoftThreshold (Supplementary Figure S3A). Then, we generated similarity matrices based on the selected soft-threshold power; the adjacency matrix was first calculated and then converted into a topological overlap matrix (TOM) to minimize noise and spurious association (Supplementary Figure S3B). From the TOM, gene modules were identified where hierarchical clustering of the genes was done based on the TOM dissimilarity measure (Supplementary Figure S3C). Using the cutreeDynamic function, we obtained 22 significant gene modules and then assigned a unique color label to each module (note: smaller modules were merged) (Supplementary Figure S3D). After characterizing the 22 gene modules (Supplementary Figures S4, S5), we analyzed the module-trait relationship to find how each of the 22 gene modules correlated to the two phenotype traits (i.e., healthy control and PAD). From the 22 gene modules, the turquoise module (Supplementary File S2C) and pink module (Supplementary File S2D) were most characteristic of the PAD phenotype based on module-trait correlation analysis (Supplementary Figure S6). We then assessed the correlations between module membership (MM) and gene significance (GS) for PAD and found the turquoise and pink modules to show the highest correlations (0.49 and 0.48, respectively) to the PAD phenotype (Supplementary Figure S7).

### PPI Network Analysis

PPI pairs were predicted for the 680 DEGs using the STRING database, with a minimum required interaction score of 0.700 (high confidence). The PPI network consisted of 444 nodes (denoting genes) and 2,398 edges (denoting interactions between genes) (Supplementary File S4). Degrees of the PPI network nodes obeyed exponential distribution (*r*-squared = 0.849), indicating it is a scale-free network. The hub molecules of the PPI network were the nodes with the highest degrees; the top-ranking hub nodes were the MAC-1 subunit integrin-α-M (ITGAM/CD11B, degree = 64), the MAC-1 subunit integrin-β-2 (ITGB2/CD18, degree = 59), formyl-peptide receptor type 2 (FPR2, degree = 56), protein tyrosine phosphatase receptor-type C (PTPRC/CD45, degree = 51), and spleen tyrosine kinase (SYK, degree = 50) (Supplementary File S4). Notably, four of the top five PPI-derived hub nodes overlapped with the WGCNA-derived turquoise module (ITGAM/CD11B, ITGB2/CD18, PTPRC/CD45, and SYK), suggesting some convergence between gene-level correlations and protein-level interactions in PAD. The other significant interactions from the PPI network were CDK1 interacting with PLK1 and FOS, LPAR3 interacting with FOS and SAA1, and FOS further interacting with RLN3 (relaxin 3). These proteins and interactions likely play key roles in the PAD phenotype.

**FIGURE 1:**
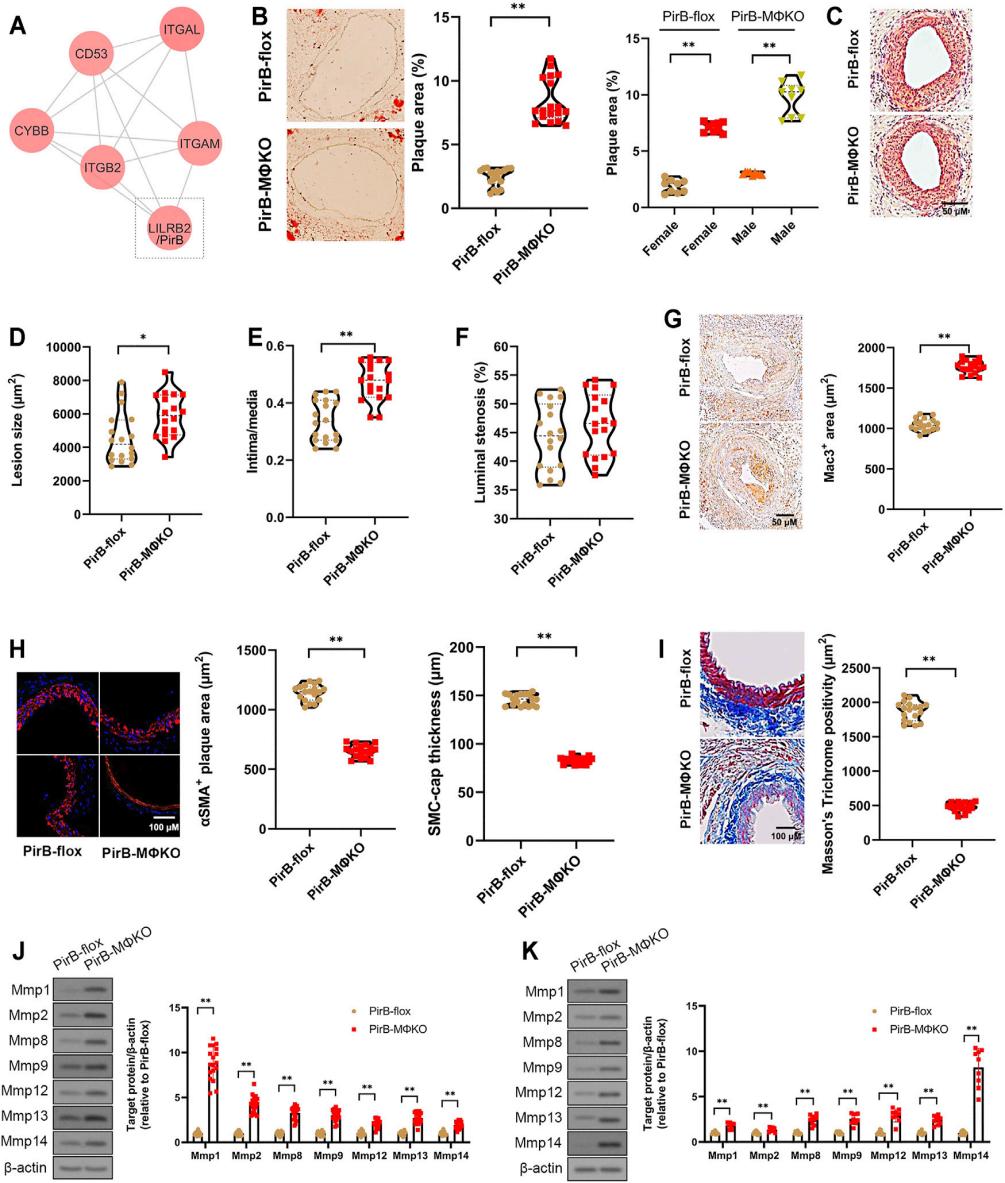
Aggravated peripheral atherogenesis and plaque vulnerability observed in PirB^MΦKO^ mice. **(A)** Bioinformatic identification of LILRB2/PirB as a key MAC-1 protein interactor in PAD. See Supplementary Figures S1–S8 for full description of results. **(B–F)** *PirB* ^flox^ mice (*n* = 9 males, 9 females) and *PirB* ^MΦKO^ mice (*n* = 9 males, 9 females) were fed on a high-fat diet (HFD) for 8 weeks and subjected to femoral arterial cuff placement during the last 2 weeks. **(B)** Oil Red O-positive lesion area as a percentage of total femoral artery surface area (overall and segregated by sex); *p < 0.05, **p < 0.01 [Mann-Whitney U test and non-parametric two-way ANOVA with Kruskal-Wallis test, respectively], **(C)** femoral artery cross-sections stained with hematoxylin and eosin (H&E) showing atherosclerotic plaques, **(D)** lesion sizes, **(E)** intima/media ratios, and **(F)** luminal stenosis rates. *p < 0.05, **p < 0.01 [Mann-Whitney U test]. **(G–I)** Immunostaining of murine femoral artery cross-sections for **(G)** Mac3-positive areas, **(H)** α-smooth muscle actin (αSMA)-positive areas and SMC-positive fibrous cap thickness, and **(I)** Masson’s trichome-positive staining for collagen content. *p < 0.05, **p < 0.01 [Mann-Whitney U test]. **(J, K)** Western blotting analysis of matrix metalloproteases (Mmps) in **(J)** murine femoral artery tissue lysates and **(K)** cultured murine peritoneal macrophages. *p < 0.05, **p < 0.01 [Student’s *t*-test]. All *in vitro* experiments: *n* = 3 biological replicates × 3 technical replicates. The horizontal lines of the boxplot indicate the median, 25th percentile, and 75th percentile; the whiskers indicate the minimum and maximum values.

The MCODE plug-in in Cytoscape was used for identifying the significant protein clusters within the PPI network. A total of 14 clusters were obtained; however, only four clusters with a score greater than ten were considered for analysis (Supplementary Figure S8). Cluster 1 (score = 28.00) consisted of 28 nodes and 378 edges, cluster 2 (score = 19.24) consisted of 22 nodes and 202 edges, cluster 3 (score = 13.69), had 13 nodes and 89 edges, and cluster 4 (score = 13.47) had 16 nodes and 101 edges (Supplementary Figure S8). As the top-ranking PPI hub nodes (the MAC-1 subunits ITGAM and ITGB2) were both located in cluster 2 and the leukocyte integrin MAC-1 has a well-recognized role in atherosclerosis (Aziz et al., 2017; Ramos et al., 2018), we chose to focus on MAC-1 protein interactors within cluster 2 to identify key regulators in PAD (Figure 1A; Supplementary File S4). Based on our ranking analysis of MAC-1 protein interactors within cluster 2, we identified the inhibitory monocyte/macrophage receptor LILRB2 as a key potential regulator of MAC-1 and chose this protein for further analysis.

### Creation and Characterization of *PirB* ^MΦKO^ Mice

LILRB2 is not expressed in mice; however, the murine monocyte/macrophage receptor PirB is LILRB2’s closest homologue and functions in an analogous manner in murine macrophages (Matsushita et al., 2011; Zheng et al., 2012). In order to analyze LILRB2/PirB’s role in PAD, we created myeloid-specific *PirB*-null mice (*Lyz* ^Cre/+^;*PirB* ^flox/flox^; *Apoe* ^−/−^), hereinafter termed *PirB* ^MΦKO^ mice, by crossbreeding *Lyz* ^Cre/+^ mice with *PirB* ^flox/flox^;*Apoe* ^−/−^ mice on a C57Bl/6 background. *PirB* ^flox/flox^;*Apoe* ^−/−^ mice (hereinafter *PirB* ^flox^) mice served as experimental controls. The offspring did not show abnormalities in development or morphology, and their genotype segregation followed the predicted Mendelian frequency. Analyzing PirB protein expression in macrophages and neutrophils, *PirB* ^flox^ neutrophils showed low levels of PirB protein expression relative to that of *PirB* ^flox^ macrophages (Supplementary Figure S9A), suggesting that PirB is preferentially expressed in macrophages. We found knockout in both cell types in *PirB* ^MΦKO^ mice (Supplementary Figure S9A), consistent with cre-recombinase selective expression by myeloid cells (Clausen et al., 1999; Ribas et al., 2011; Park S.-H. et al., 2012).

Similar to LILRB2, murine PirB is known to bind to major histocompatibility class I (MHC-I) and angiopoietin-like-protein 2 (Angptl2) ligands (Matsushita et al., 2011; Zheng et al., 2012). As anti-LILRB2 blockade suppresses anti-atherogenic Akt signaling in human macrophages (Vergadi et al., 2017; Chen et al., 2018), we hypothesized that PirB deficiency should also suppress Akt signaling in murine macrophages. Indeed, PirB knockout uniformally downregulated Akt phosphorylation in peritoneal macrophages under control, MHC-I, and Angptl2-treated conditions (Supplementary Figure S9B). Additionally, the dose-dependent increase in Akt phosphorylation by recombinant MHC-I β2M was uniformally abolished by PirB knockout (Supplementary Figure S9C). In order to stimulate intracellular Akt signaling, MHC-I binding autophosphorylates the immunoreceptor tyrosine-based inhibition motif (ITIM) domain of PirB that recruits and binds to Src homology 2 domain-containing phosphatase 1 (Shp1) (Van Der Touw et al., 2017; Azcutia et al., 2018). Accordingly, our anti-PirB immunoprecipitation (IP) experiments in *PirB* ^flox^ macrophages led to effective pull-down of p-Shp1, while anti-PirB IP in PirB^MΦKO^ macrophages displayed negligible p-Shp1 pulldown (Supplementary Figure S9D). These results indicate that PirB^MΦKO^ abolishes the PirB/p-Shp1 interaction and inhibits downstream Akt signaling in macrophages.

Previous evidence suggests that pro-inflammatory Ly6C^hi^ monocytes (CD11b^+^/CD90^−^/B220^−^/CD49b^−^/NK1.1^−^/Ly6G^−^/Ly6C^hi^ cells) adhere to activated endothelium, infiltrate atherosclerotic lesions, and transform into lesional macrophages (Swirski et al., 2007). Notably, Ly6C^hi^ monocyte counts are dramatically increased in hyperlipidemic *Apoe* ^−/−^ mice (Swirski et al., 2007). Consistent with previous results (Swirski et al., 2007; Murphy et al., 2011), we found that HFD enhanced total monocyte and pro-inflammatory Ly6C^hi^ monocyte counts (Supplementary Table S2). Notably, *PirB* ^MΦKO^ and *PirB* ^flox^ mice showed no significant differences in total or Ly6C^hi^ monocyte counts (Supplementary Table S2). Moreover, *PirB* ^MΦKO^ and *PirB* ^flox^ mice showed no significant differences in cholesterol and pro-inflammatory cytokine levels (Supplementary Table S3). These results indicate that *PirB* ^MΦKO^ has no significant effect on circulating monocytes and pro-inflammatory markers.

### Macrophage *PirB* Knockout Increases Peripheral Atherogenesis and Plaque Vulnerability *In Vivo*

After 2 weeks of cuff-induced atherogenesis in HFD-fed mice, the mice were assessed for femoral atherosclerotic lesions. A significant increase was observed in the Oil Red O positive lesion area in the femoral arteries in *PirB* ^MΦKO^ mice (Figure 1B). The effect was confirmed in both sexes indicating that PirB deficiency’s effects on peripheral atherosclerosis is not sex-specific (Figure 1B). Consistent with the increased atherogenesis, we also observed significant increases in femoral plaque sizes and intima/media ratios in *PirB* ^MΦKO^ mice (Figures 1C–E); however, we did not observe significant differences in overall luminal stenosis between *PirB* ^flox^ and *PirB* ^MΦKO^ mice (Figure 1F). As expected, we noted increases in Mac3-positive cell (macrophage) content (Figure 1G) in *PirB* ^MΦKO^ plaques. *PirB* ^MΦKO^ plaques also showed lower α-SMA-positive cell (SMC) content (Figure 1H), more thinning in SMC-positive fibrous caps (Figure 1H), and reduced collagen content by Masson’s trichrome stain (Figure 1I). Since there was a reduction in collagen content, we wanted to assess if this could be due to increased matrix metalloproteinase (Mmp) activity. Significant increases in protein levels of Mmp-1, Mmp-2, Mmp-8, Mmp-9, Mmp-12, Mmp-13, and Mmp-14 were observed in femoral plaques from *PirB* ^MΦKO^ mice ((Figure 1J). Validating our findings, we also observed increased expression of Mmp-1, Mmp-2, Mmp-8, Mmp-9, Mmp-12, Mmp-13, and Mmp-14 in *PirB* ^MΦKO^ peritoneal macrophages *in vitro* (Figure 1K).

Since our results in *PirB* ^MΦKO^ indicated enhanced peripheral plaque vulnerability, we evaluated the other indicators of femoral artery plaque vulnerability. Femoral artery cross-sections were stained with Carstairs’ (Carstairs, 1965) and Verhoeff–Van Gieson stain (Kozaki et al., 2002) to identify disruptions in fibrous cap, intra-plaque hemorrhage, deposition of fibrin, or medial elastin breaks (Supplementary Figure S10). If any of these features were observed in three consecutive sections, we considered it a positive sign of plaque vulnerability. *PirB* ^MΦKO^ mice showed enhanced plaque vulnerability with higher percentages of intraplaque hemorrhage (11/18 *PirB* ^MΦKO^ mice vs. 2/18 *PirB* ^flox^ mice, *p* < 0.01; Supplementary Table S4) and medial elastin breaks (6/18 *PirB* ^MΦKO^ mice vs. 0/18 *PirB* ^flox^ mice, *p* < 0.01; Supplementary Table S4). This data suggests that *PirB* ^MΦKO^ plaques are more unstable than *PirB* ^flox^ control plaques.

### Macrophage *PirB* Knockout Enhances Macrophage Pro-Inflammatory Response *In Vitro*

**FIGURE 2:**
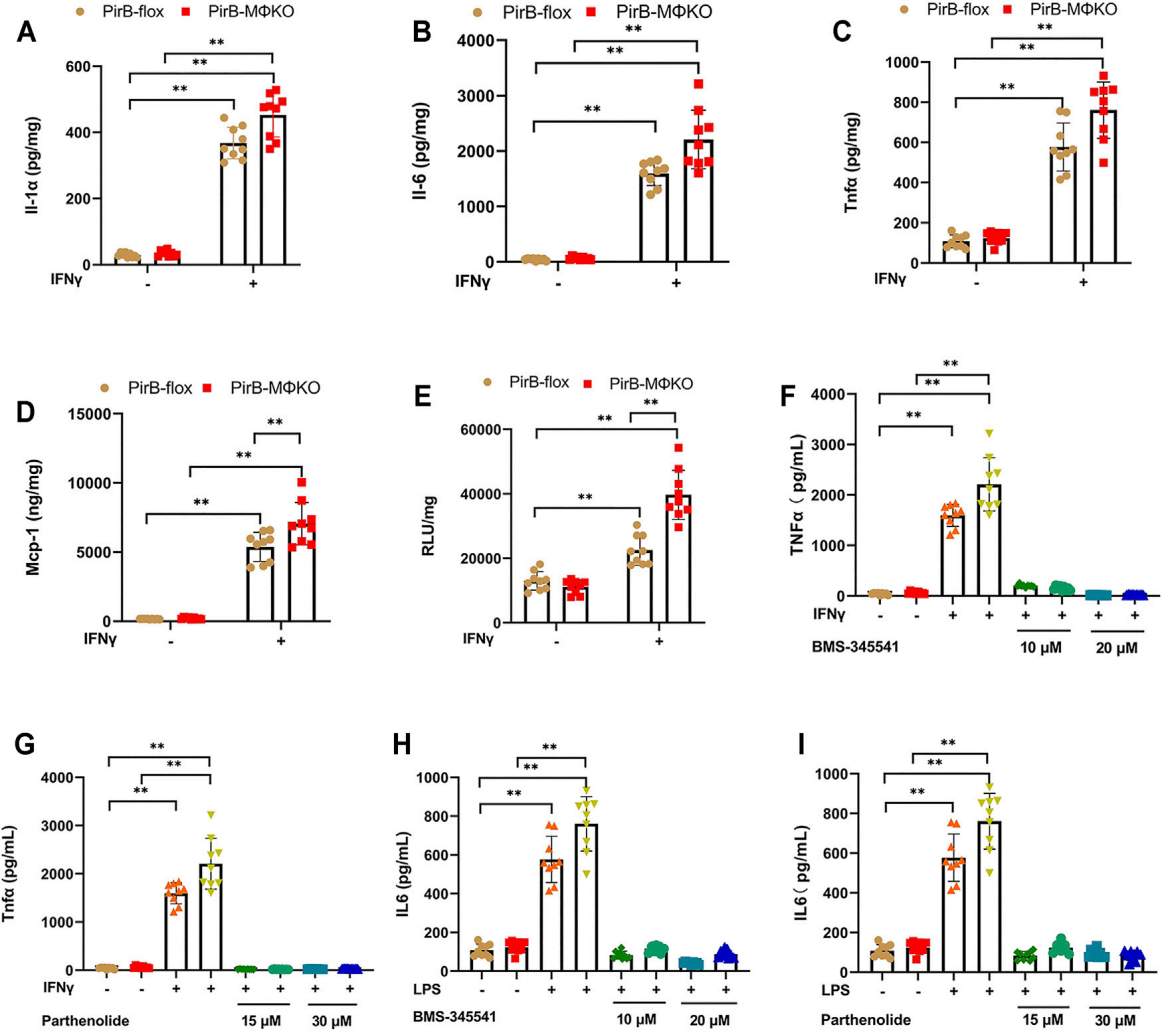
Macrophage *PirB* knockout enhances macrophage pro-inflammatory response *in vitro*. **(A–D)** The response of overnight cultured *PirB* ^flox^ and *PirB* ^MΦKO^ peritoneal macrophages to interferon-γ (IFNγ). Non-adherent cells were removed from the culture before priming adhered cells with IFNγ, and the conditioned cells were collected after 24 h. Adherent peritoneal macrophages were assessed for the secretion of **(A)** interleukin (Il)-1α, **(B)** Il-6, **(C)** tumor necrosis factor-α (Tnfα), and **(D)** monocyte chemoattractant protein-1 (Mcp-1) by ELISA. **(E)** Cultured macrophage lysates were assessed for nuclear factor-κB (NFκB) DNA binding activity before and after priming with IFNγ for 3 h **(F–I)** Macrophages were exposed to a directed dose of either BMS-345541 or parthenolide for 1 h before activation by IFNγ or LPS. **(F,G)** Il-6 and **(H, I)** Tnfα production in conditioned media were assessed after 24 h by ELISA. *p < 0.05, **p < 0.01 [two-way ANOVA with Bonferroni post-hoc test]. All *in vitro* experiments: *n* = 3 biological replicates × 3 technical replicates.

To evaluate the pro-inflammatory response in *PirB* ^MΦKO^ macrophages, we tested thioglycollate-elicited peritoneal macrophages for interleukin (Il)-1α, Il-1β, Il-6, Tnfα, and Mcp-1 levels. Except for Il-1β, the production of all inflammatory markers was detectable. *PirB* ^MΦKO^ macrophages showed a significantly increased secretion of Il-1α, Il-1β, Il-6, Tnfα, and Mcp-1 on exposure to interferon-γ (IFNγ) (Figures 2A–D).

As nuclear factor-κB (NFκB) activation enhances inflammatory cytokine and chemokine production, we analyzed NFκB DNA binding activity. We observed a significantly greater NFκB DNA binding activity in *PirB* ^MΦKO^ macrophages that was further augmented by IFNγ (Figure 2E). Further assessing the participation of NFκB, we exposed *PirB* ^MΦKO^ cells to the NFκB inhibitors BMS-345541 and parthenolide. Both BMS-345541 and parthenolide totally eliminated the increases in IFNγ-driven Il-6 production (Figures 2F,G) and IFNγ-driven Tnfα production (Figures 2H,I). This evidence supports the role of NFκB-dependent cytokine production in the pro-inflammatory response of *PirB* ^MΦKO^ macrophages to IFNγ.

### Macrophage *PirB* Knockout Promotes Classical M1 Polarization *via* a Shp1-Dependent Mechanism *In Vitro*

**FIGURE 3:**
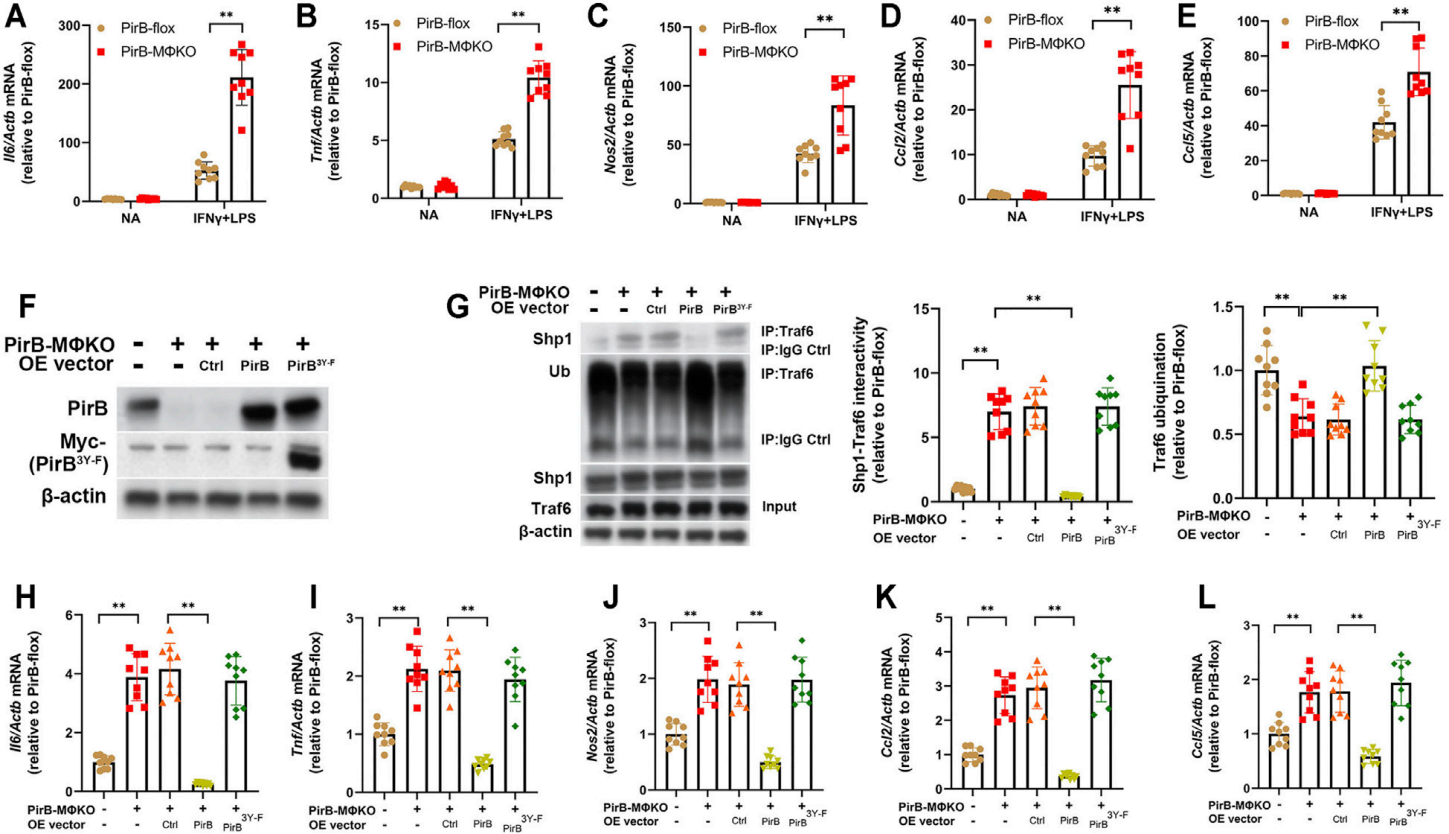
Macrophage *PirB* knockout promotes classical M1 macrophage polarization via a Shp1-dependent mechanism *in vitro*. **(A–E)** Adherent *PirB* ^flox^ and *PirB* ^MΦKO^ peritoneal macrophages were either non-activated (NA) or exposed to IFNγ and LPS for M1 activation. qPCR analysis for the M1 activation markers **(A)** *Il6*, **(B)** *Tnf*, **(C)** *Nos2*, **(D)** *Ccl2*, and **(E)** *Ccl5*. **(F–L)** Adherent *PirB* ^MΦKO^ macrophages were transfected with WT PirB or a Myc-tagged PirB ITIM domain tyrosine phosphorylation site mutant (PirB^3Y-F^). **(F)** Western blotting analysis of PirB and Myc-tagged PirB^3Y-F^. **(G)** Immunoprecipitation (IP) with an anti-Traf6 antibody (or IgG control) and densitometric analysis of Shp1-Traf6 binding and Traf6 ubiquination. qPCR analysis for the M1 activation markers **(H)** *Il6*, **(I)** *Tnf*, **(J)** *Nos2*, **(K)** *Ccl2*, and **(L)** *Ccl5*. *p < 0.05, **p < 0.01 [**(A–E)** two-way ANOVA with Bonferroni post-hoc test; **(G–L)** one-way ANOVA with Bonferroni post-hoc test]. All *in vitro* experiments: *n* = 3 biological replicates × 3 technical replicates.

Having observed an increased pro-inflammatory response in *PirB* ^MΦKO^ macrophages, we evaluated the role of PirB deficiency in the polarization of macrophages. Upon exposure to IFNγ and lipopolysaccharide (IFNγ+LPS), there was a higher degree of classical activation (M1) in *PirB* ^MΦKO^ macrophages as compared with *PirB* ^flox^ macrophages. This was confirmed by enhanced expression of M1 marker genes (*Il6*, *Tnf*, *Nos2*, *Ccl2*, and *Ccl5*) (Figures 3A–E). In contrast, *PirB* ^MΦKO^ abrogated the rise in alternative activation (M2) markers (*Arg1*, *Mrc1*, and *Pparg*) in response to IL-4 as observed with *PirB* ^flox^, indicating that PirB deficiency reduces M2 activation (Supplementary Figure S11A–C).

Our aforedescribed anti-PirB IP experiments indicated that *PirB* ^MΦKO^ abolishes the PirB/p-Shp1 interaction in macrophages (Supplementary Figure S9D). As Shp1’s binding to the adaptor protein Traf6 reduces Traf6 ubiquination and consequent NFκB-mediated inflammatory cytokine production in macrophages (Yan et al., 2012), we hypothesized that PirB deficiency may promote M1 macrophage polarization through reducing Shp1-Traf6 binding and promoting Traf6 ubiquination. To test this, we employed *PirB* ^MΦKO^ macrophages with or without ectopic overexpression of WT PirB or a PirB ITIM domain tyrosine phosphorylation site mutant (PirB^3Y-F^) that is incapable of recruiting Shp1 (Lu et al., 2018) (Figure 3F). We found that overexpression of WT PirB, but not the PirB^3Y-F^ mutant, reduced Shp1-Traf6 binding (Figure 3G), rescued Traf6 ubiquination to baseline levels (Figure 3G), and abrogated the upregulation of M1 marker genes (*Il6*, *Tnf*, *Nos2*, *Ccl2*, and *Ccl5*) produced by *PirB* ^MΦKO^ (Figures 3H–L). This evidence suggests that PirB deficiency favors a shift toward M1 polarization through a Shp1-dependent mechanism.

### Macrophage *PirB* Knockout Promotes Classical M1 Polarization Over Alternative M2 Polarization Following oxLDL Exposure *In Vitro*

**FIGURE 4:**
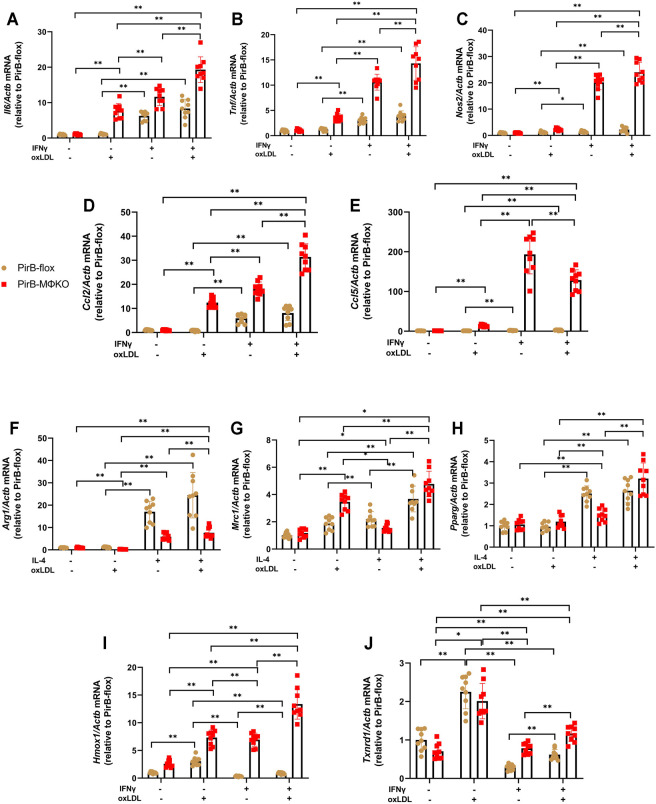
Macrophage *PirB* knockout promotes classical M1 macrophage polarization over alternative M2 macrophage polarization following oxLDL exposure *in vitro*. Adherent *PirB* ^flox^ and *PirB* ^MΦKO^ peritoneal macrophages were treated with **(A–E, I, J)** IFNγ or **(F–H)** IL-4 for 6 h and then exposed to oxLDL for 18 h qPCR analysis of the M1 activation markers **(A)** *Il6*, **(B)** *Tnf*, **(C)** *Nos2*, **(D)** *Ccl2*, and **(E)** *Ccl5*; **(F–H)** M2 activation markers **(F)** *Arg1*, **(G)** *Mrc1*, and **(H)** *Pparg;* and oxLDL-induced (Mox) activation markers **(I)** *Hmox1* and **(J)** *Txnrd1*. *p < 0.05, **p < 0.01 [two-way ANOVA with Bonferroni post-hoc test]. All *in vitro* experiments: *n* = 3 biological replicates × 3 technical replicates.

Several reports suggest that oxLDL and its components can alter the polarization of macrophages (Kadl et al., 2010; Wiesner et al., 2010; Van Tits et al., 2011; Park Y. M. et al., 2012). We evaluated if PirB deficiency affects M1 and M2 marker gene expression following oxLDL exposure in IFNγ-treated or IL-4-treated macrophages. M1 marker (*Il6*, *Tnf*, *Nos2*, *Ccl2*, and *Ccl5*) expression remained unchanged in IFNγ-treated *PirB* ^flox^ macrophages upon oxLDL exposure (Figures 4A–E). However, in IFNγ-treated *PirB* ^MΦKO^ macrophages, oxLDL further increased the levels of *Tnf*, *Nos2*, *Il6*, and *Ccl2* but reduced *Ccl5* levels. Furthermore, M2 markers *Arg1* and *Pparg* remained unchanged, while *Mrc1* was upregulated, in IL-4-treated *PirB* ^flox^ macrophages upon oxLDL exposure (Figures 4F–H). However, *PirB* knockout only abrogated the influence of oxLDL on *Arg1* (but not *Mrc1* or *Pparg*) in IL-4-treated macrophages. This indicates that PirB deficiency affects the macrophage-polarizing effects of oxLDL.

Several reports suggest that oxLDL and its components can induce Mox polarization (Kadl et al., 2010; Wiesner et al., 2010; Van Tits et al., 2011; Park Y. M. et al., 2012). Upon exposure to oxLDL, macrophages have shown to have enhanced expression of the Mox signature genes hemeoxygenase-1 (*Hmox1*) and thioredoxin reductase-1 (*Txnrd1*), leading to Mox phenotypic polarization of the macrophages (Kadl et al., 2010). In *PirB* ^flox^ macrophages, the expression of *Hmox1* and *Txnrd1* was suppressed by IFNγ; but in *PirB* ^MΦKO^ macrophages, IFNγ significantly upregulated *Hmox1* (Figure 4I). *Txnrd1* levels, even though suppressed in *PirB* ^MΦKO^, were still lower as compared to *PirB* ^flox^ macrophages (Figure 4J). Thus, *Txnrd1* expression was enhanced in *PirB* ^MΦKO^ macrophages as compared with *PirB* ^flox^ macrophages.

### Macrophage *PirB* Knockout Enhances Macrophage Efferocytosis *In Vitro*

Efferocytosis is the phagocytic clearance of apoptotic cells and is a crucial anti-inflammatory process performed by macrophages (Elliott et al., 2017). Evaluating the effect of PirB deficiency on mRNA expression of efferocytosis marker genes (*Anxa1*, *Gas6*, *C1qa*, *Mertk*, and *Mfge8*), we observed that there were changes in their levels in both activated and non-activated macrophages. In *PirB* ^flox^ macrophages, M1 activation by IFNγ+LPS leads to repression of efferocytosis marker genes, while M2 activation by IL-4 had no influence on *Anxa1*, *C1qa*, and *Mertk* expression but downregulated *Gas6* and *Mfge8* levels (Supplementary Figures S12A–E). In PirB^flox^ macrophages, oxLDL did not show any noticeable effect except on *Mertk* upregulation. However, oxLDL enhanced levels of *Anxa1* and *Mfge8* but repressed *C1qa* expression in *PirB* ^MΦKO^ macrophages (Supplementary Figures S12A–E).

We next evaluated the effect of PirB deficiency on macrophage efferocytosis of CMFDA-labeled apoptotic thymocytes. In *PirB* ^flox^ macrophages, M1 activation by IFNγ+LPS leads to repression of efferocytosis activity as measured by short-term CMFDA+ apoptotic thymocyte uptake (Supplementary Figure S12F) as well as phagocytic indices from longer-term single-feeding and double-feeding experiments (Supplementary Figures S12G,H). In contrast, M2 activation by IL-4 had no influence on these efferocytosis parameters (Supplementary Figures S12F–H). In *PirB* ^flox^ macrophages, oxLDL did not show any noticeable effect on these efferocytosis parameters. However, oxLDL enhanced efferocytosis activity in *PirB* ^MΦKO^ macrophages (Supplementary Figures S12F–H). Thus, PirB deficiency appears to promote efferocytosis activity under oxLDL-treated conditions.

### Macrophage *PirB* Knockout Downregulates Cholesterol Efflux *In Vitro*

**FIGURE 5:**
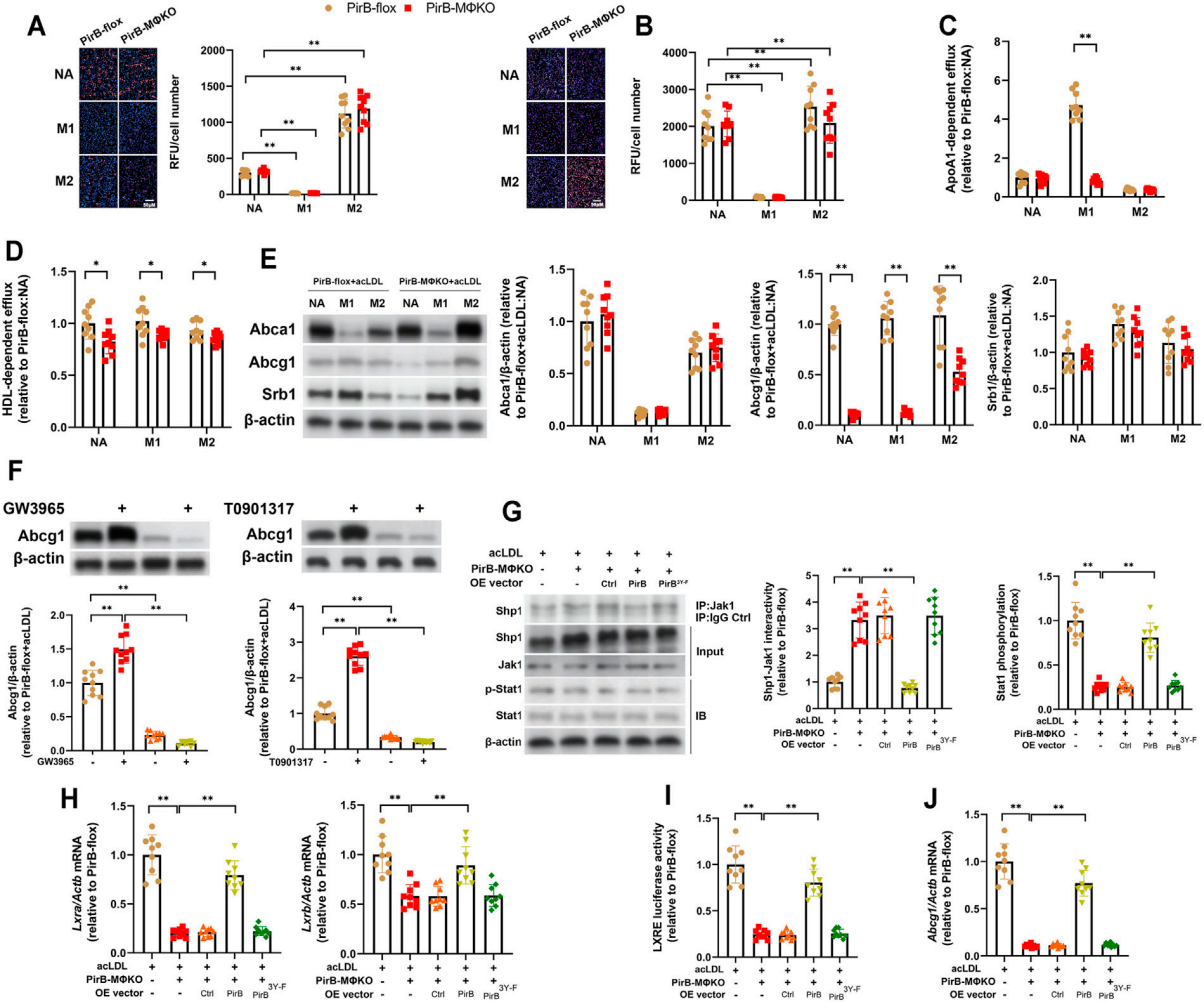
Macrophage *PirB* knockout downregulates cholesterol efflux *in vitro*. **(A,B)** Adherent *PirB* ^flox^ and *PirB* ^MΦKO^ peritoneal macrophages were either non-activated (NA) or exposed to oxLDL and IFNγ and LPS for M1 activation or IL-4 for M2 activation for 24 h and exposed to **(A)** acetylated LDL (acLDL) or **(B)** oxidized LDL (oxLDL) for 48 h. Oil Red O staining was quantified as the relative fluorescent unit (RFU) per cell. **(C,D)** Macrophages were exposed to acLDL and 3H-cholesterol for 24 h; **(C)** apolipoprotein AI (ApoAI)- or **(D)** high-density lipoprotein (HDL)-dependent 3H-cholesterol efflux were analyzed for 24 h. **(E)** Macrophages were exposed to acLDL for 24 h followed by Western blotting analysis of Abca1, Abcg1, and Srb1 protein expression. **(F)** AcLDL-exposed macrophages were treated with GW3965 (1 µM) or T0901317 (1 µM) for 48 h and assessed for *Abcg1* expression. **(G–J)** Adherent *PirB* ^MΦKO^ macrophages that had been transfected with WT PirB or a Myc-tagged PirB ITIM domain tyrosine phosphorylation site mutant (PirB^3Y-F^) were exposed to acLDL for 24 h. **(G)** Immunoprecipitation (IP) with an anti-Jak1 antibody (or IgG control) and standard immunoblotting (IB) followed by densitometric analysis of Shp1-Jak1 binding and Stat1 phosphorylation. **(H)** qPCR analysis of *Lxra* and *Lxrb* expression. **(I)** Analysis of LXRE luciferase activity. **(J)** qPCR analysis of *Abcg1* expression. *p < 0.05, **p < 0.01 [**(A–F)** two-way ANOVA with Bonferroni post-hoc test; **(G–J)** one-way ANOVA with Bonferroni post-hoc test]. All *in vitro* experiments: *n* = 3 biological replicates × 3 technical replicates.

Macrophage accumulation of modified LDLs produces atherogenic foam cells (Orekhov, 2018). Here, we tested for intracellular lipid accumulation from 48-h exposure to oxLDL and acLDL following M1 activation by IFNγ+LPS or M2 activation by IL-4. M1 cells internalized less lipid content relative to NA or M2 cells (Figures 5A,B), while M2 cells accumulated higher lipid content relative to NA cells following exposure to acLDL (Figure 5A). Notably, PirB knockout did not affect lipid accumulation, regardless of NA/M1/M2 status or lipid exposure (Figures 5A,B). Cholesterol efflux also plays a role in macrophagic lipid incorporation, we tested activated macrophages for cholesterol efflux activity. PirB knockout downregulated apoAI-dependent efflux in M1 macrophages (Figure 5C) and HDL-dependent efflux in NA, M1, and M2 macrophages (Figure 5D).

### Macrophage *PirB* Knockout Downregulates the Major Cholesterol Transporter Abcg1 *via* a Shp1-dependent Mechanism *In Vitro*

To further investigate, we assessed the protein expression of the major cholesterol transporters ATP binding cassette subfamily A member 1 (Abca1), ATP binding cassette subfamily G member 1 (Abcg1), and scavenger receptor class B member 1 (Srb1) in acLDL-exposed *PirB* ^flox^ and *PirB* ^MΦKO^ macrophages (Tang et al., 2012). Only Abcg1 levels were decreased in *PirB* ^MΦKO^ macrophages (Figure 5E). As Abcg1 is positively regulated by LXRα/β signaling, we treated the acLDL-exposed *PirB* ^flox^ and *PirB* ^MΦKO^ macrophages with the LXRα/β agonists GW3965 or T0901317 (Magida and Evans, 2018). Notably, both LXRα/β agonists induced Abcg1 upregulation in *PirB* ^flox^ but not *PirB* ^MΦKO^ macrophages (Figure 5F). Thus, LXRα/β-driven upregulation of Abcg1 is negatively regulated by PirB knockout. The PirB interactor Shp1 has been shown to negatively regulate Jak1-Stat1 signaling, an upstream regulator of LXRα/β expression in macrophages (Kim et al., 2016; Abram and Lowell, 2017). Therefore, we hypothesized that PirB knockout may downregulate LXRα/β expression and resulting Abcg1 expression in acLDL-exposed macrophages through the Shp1-Jak1-Stat1 axis. To test this, we exposed the aforedescribed *PirB* ^MΦKO^ macrophages with or without ectopic overexpression of WT PirB or the PirB^3Y-F^ mutant (Figure 3F) to acLDL and conducted a series of experiments. We found that overexpression of WT PirB, but not the PirB^3Y-F^ mutant, rescued Shp1-Jak1 binding (Figure 5G), Stat1 phosphorylation (Figure 5G), LXRα/β expression (Figure 5H), LXRE activity (Figure 5I), and Abcg1 downregulation (Figure 5J) produced by *PirB* ^MΦKO^. This evidence indicates that PirB deficiency promotes LXRα/β-driven Abcg1 downregulation in a Shp1-dependent manner.

### Macrophage *PirB* Knockout Enhances Monocyte Recruitment, Macrophage Efferocytosis, and Inflammatory Marker Expression in Femoral Plaques *In Vivo*

As we had observed an upsurge in Mac3-positive plaques in *PirB* ^MΦKO^ mice, we wanted to assess several key indicators of macrophage activity in atherosclerotic lesions, namely: 1) recruitment of monocytes, 2) intra-lesion macrophage proliferation, 3) intra-lesion macrophage apoptosis, and 4) inflammatory marker expression (Robbins et al., 2013; Randolph, 2014).

**FIGURE 6:**
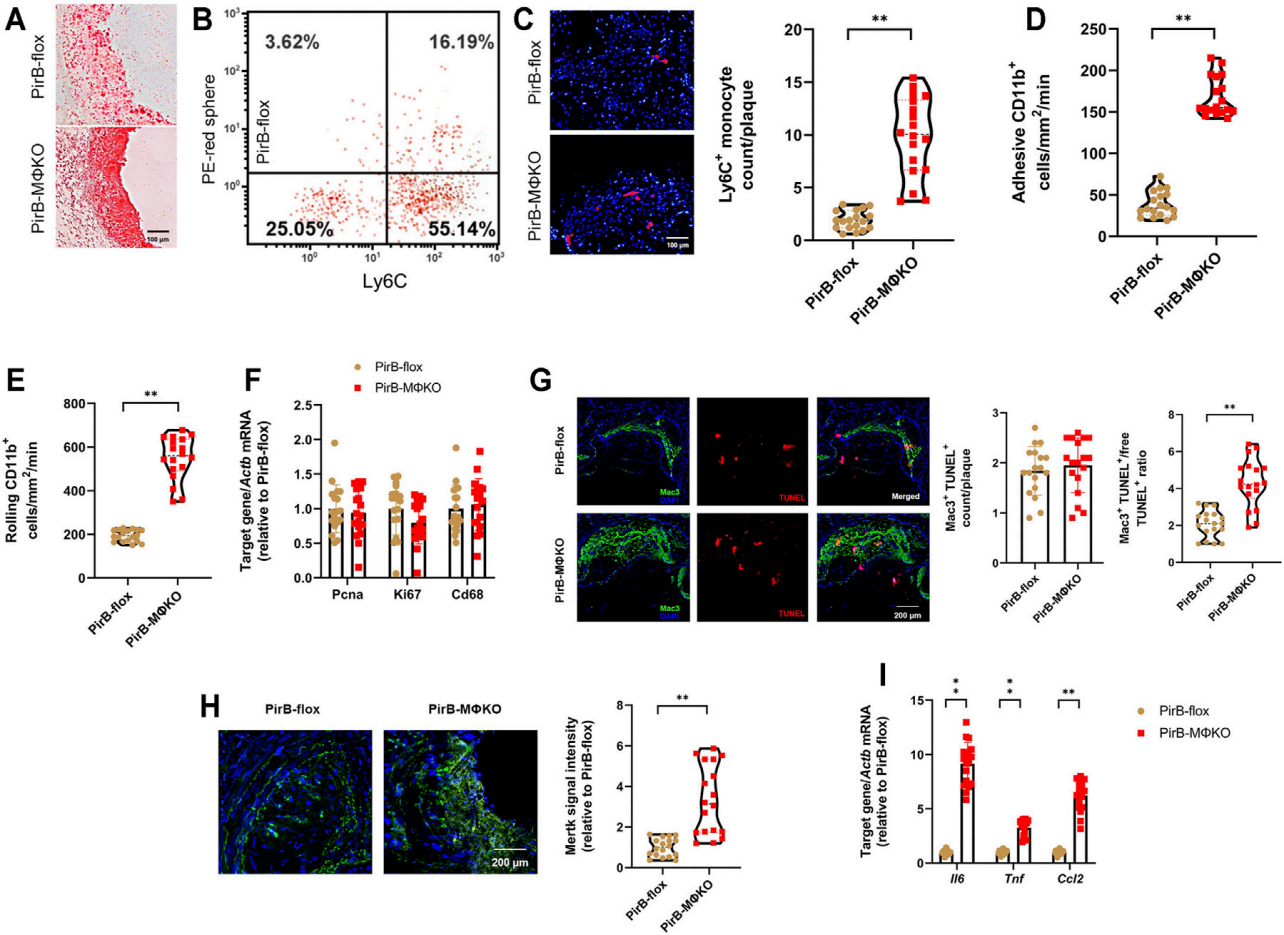
Macrophage *PirB* knockout enhances monocyte recruitment, macrophage efferocytosis, and inflammatory marker expression in femoral plaques *in vivo*. *PirB* ^flox^ mice (*n* = 9 males, 9 females) and *PirB* ^MΦKO^ mice (*n* = 9 males, 9 females) fed on a high-fat diet (HFD) for 12 weeks and subjected to femoral arterial cuff placement during the last 2 weeks. Vehicle or clodronate liposomes were administered a day before administration of red microspheres. Mice were administered with red microspheres 3 and 7 days before sacrifice. Monocyte infiltration within the plaque areas was observed from flow cytometry analysis of red microspheres. **(A)** Oil Red O-stained serial frozen sections of the femoral artery. **(B)** Flow cytometry analysis of circulating monocytes following clodronate and red microsphere administration, showing presence of both Ly6C^hi^ and Ly6C^lo^ monocyte populations. Leukocytes were stained with anti-CD115-biotin-avidin-eFluor450, anti-CD11b-AlexaFluor488, and anti-Ly6C-AlexaFluor700 for monocyte gating. The *x*-axis shows Ly6C-positivity and the *y*-axis (detected by the PE filter) shows red microsphere-positivity. Percentages within the monocyte-gated population are indicated. **(C)** DAPI-stained serial frozen section of the femoral artery following clodronate and red microsphere administration. DAPI-stained nuclei are blue, while red microspheres are red. Number of red microspheres per plaque are quantified. **(D)** Cell adhesion and **(E)** rolling of CD11b^+^Ly6C^+^ inflammatory monocytes on the luminal surface of endothelium by intravital microscopy. **(F)** qPCR analysis of the cell proliferation markers *Pcna* and *Ki67* within the macrophage-rich immunostained plaques isolated by laser capture microdissection (LCM). qPCR analysis of *Cd68* levels confirmed equivalent sections of macrophage-rich areas. **(G)** Terminal deoxynucleotidyl transferase dUTP nick end labeling (TUNEL) staining (red), Mac3 staining (green), and DAPI staining (blue) enabled colocalization of TUNEL- and Mac3-positive macrophages within PFA-fixed, paraffin-embedded sections of femoral arteries. Quantification of macrophage apoptosis (Mac3^+^TUNEL^+^) and the Mac3^+^TUNEL^+^ cells-to-free TUNEL^+^ cell ratio. **(H)** Mertk staining (green) and DAPI staining (blue) within PFA-fixed, paraffin-embedded sections of femoral arteries. Quantification of Mertk staining. **(I)** qPCR analysis of the inflammatory markers *Il6*, *Tnf*, and *Ccl2* within the macrophage-rich immunostained plaques isolated by LCM. The horizontal lines of the boxplot indicate the median, 25th percentile, and 75th percentile; the whiskers indicate the minimum and maximum values. *p < 0.05, **p < 0.01 [**(C–E, G (right), H)** Mann-Whitney U test; **(F, G (left), I)** Student’s *t*-test].

For evaluating the recruitment of monocytes into femoral lesions (Figure 6A), we administered vehicle or clodronate followed by labeling of circulating monocytes by injecting red microspheres into murine tail veins. As these microspheres cannot pass through the interstitial space, all red-fluorescent/DAPI-positive cells detected within plaques by flow cytometry were considered recruited and infiltrated from the circulation. There were no detectable red-fluorescent cells (i.e., Ly6C^lo^ monocytes) with vehicle administration in either *PirB* ^flox^ or *PirB* ^MΦKO^ plaques (Higashi et al., 2016). However, due to administration of clodronate, red-fluorescent labeling was applied to both circulating Ly6C^lo^ monocytes and Ly6C^hi^ monocytes (Higashi et al., 2016) (Figure 6B). Following clodronate administration, a significantly higher number of red-fluorescent cells were detected in *PirB* ^MΦKO^ plaques (Figure 6C). This indicates that PirB deficiency promotes pro-inflammatory Ly6C^hi^ monocyte infiltration into femoral lesions (Swirski et al., 2007).

CD11b expression on leukocytes regulates leukocyte adhesion and rolling on the endothelium, an early step in atherogenesis (Woollard and Geissmann, 2010). To evaluate the role of PirB on monocyte adhesion and rolling on the endothelial luminal surface *in vivo*, CD11b^+^Ly6C^+^ inflammatory monocytes were identified in the mesenteric circulation using intravital microscopy. There was a significant rise in CD11b^+^Ly6C^+^ monocyte adhesion (Figure 6D) and rolling (Figure 6E) on the endothelial luminal surface of *PirB* ^MΦKO^ mice. Given that *PirB* ^MΦKO^ does not impact circulating total monocyte or Ly6C^hi^ monocyte counts (Supplementary Table S2), this combined evidence supports that PirB knockout enhances recruitment of CD11b^+^Ly6C^+^ monocytes into lesions.

To validate our *in vitro* findings, we finally evaluated intra-plaque macrophage proliferation and apoptosis as well as inflammatory marker expression within the macrophage-rich areas in the femoral plaques (Kelman, 1997; Ozen and Ittmann, 2005; Brizova et al., 2010). To dissect out the macrophage-rich areas, we used the SC-101447 monoclonal antibody against mouse macrophages (Foryst-Ludwig et al., 2010; Kuhn et al., 2013). These macrophage-rich areas were subjected to qPCR, which confirmed equivalent levels of *Cd68*, *Ki67*, and *Pcna* expression in *PirB* ^MΦKO^ lesions as compared with *PirB* ^flox^ lesions (Figure 6F). Although *PirB* ^MΦKO^ produced no significant differences in macrophage apoptosis (Mac3^+^TUNEL^+^) counts, it did increase the ratio of macrophage-associated TUNEL^+^ (Mac3^+^TUNEL^+^) cells-to-free TUNEL^+^ cells (Figure 6G), an indicator of efferocytosis (Yurdagul Jr et al., 2020). Accordingly, we also found enhanced staining of the efferocytosis marker Mertk in *PirB* ^MΦKO^ lesions as compared with *PirB* ^flox^ lesions (Figure 6H). With respect to inflammatory markers, we observed enhanced *Il6*, *Tnf*, and *Ccl2* levels within the macrophage-rich areas of *PirB* ^MΦKO^ lesions as compared with those of *PirB* ^flox^ lesions by qPCR (Figure 6I). These results show that PirB knockout increases recruitment of monocytes to lesions as well as intra-lesional efferocytosis and inflammation but does not significantly affect intra-lesion macrophage proliferation or apoptosis.

## Discussion

PAD is a common disease affecting patient quality of life. Although the epidemiology is known, the pathogenesis mechanism is not well elucidated. Based on an integrated bioinformatics approach, here we discovered the inhibitory monocyte/macrophage receptor LILRB2 to be strongly associated with the PAD phenotype. Although a number of reports have indicated LILRB2 expression in human macrophages (Chen et al., 2018; Van Dalen et al., 2019), its exact role with respect to molecular mechanisms underlying peripheral atherosclerosis remain elusive. To understand the role of LILRB2 in PAD, we analyzed the role of its close murine homologue PirB by generating myeloid-specific PirB knockout (*PirB* ^MΦKO^). In our myeloid-specific *PirB*-null *Apoe* ^−/−^ murine model of PAD, PirB deficiency increased macrophage recruitment to atherosclerotic plaques and increased atherosclerotic burden. Accordingly, we identified that PirB deficiency plays a key role in promoting the inflammatory response and inhibiting cholesterol efflux in macrophages *in vitro*. The present work suggests that macrophage PirB deficiency is pro-inflammatory and leads to an increase in peripheral atherosclerotic burden in mice.

In this study, the *PirB* ^MΦKO^ (*Lyz* ^Cre/+^;*PirB* ^flox/flox^ *;Apoe* ^−/−^) mice had one allele of *Lyz* ablated (Clausen et al., 1999; Ribas et al., 2011; Park S.-H. et al., 2012), whereas the *PirB* ^flox^ (*PirB* ^flox/flox^ *;Apoe* ^−/−^) mice had both *Lyz* alleles intact. *Lyz* ^Cre/+^ mice with hemizygous deficiency in *Lyz* show no evident difference from the heterozygous phenotype with respect to macrophage/monocyte biology (Clausen et al., 1999). Moreover, atherosclerosis in *Apoe* ^−/−^ mice is not influenced by compete ablation of both *Lyz* alleles (Rotzius et al., 2009). We confirmed the successful knockout of PirB in *PirB* ^MΦKO^ macrophages and neutrophils. However, PirB expression was negligible in *PirB* ^flox^ neutrophils relative to *PirB* ^flox^ macrophages, suggesting that macrophagic PirB likely plays a more important role in the *PirB* ^MΦKO^ phenotype, although a contributory role by neutrophilic PirB cannot be excluded.

Upon infiltration into a target tissue and exposure to stimuli, macrophages become activated either along the classic M1 pathway (producing a pro-inflammatory phenotype) or along the alternate M2 pathway (producing a less inflammatory, phagocytic phenotype) (Vergadi et al., 2017). Previous work in hypercholesterolemic mouse models suggest a phenotypic shift favoring M2 over M1 activation reduces atherosclerotic burden (Babaev et al., 2014; Harmon et al., 2014; Wolfs et al., 2014). Recent findings also report the existence of a continuum of macrophage polarization with M1 and M2 being extreme ends of the continuum (Mosser and Edwards, 2008; Martinez and Gordon, 2014; Xue et al., 2014). There has been mixed findings regarding the effects of PirB knockout on macrophage polarization, with some favoring M1 over M2 polarization (Ma et al., 2011; Karo-Atar et al., 2013) and some favoring the opposite (Kondo et al., 2013). Here, we found that macrophage PirB knockout upregulated pro-inflammatory M1 marker levels in a Shp1-dependent manner along with downregulating M2 marker levels. Consistently, *PirB* ^MΦKO^ robustly enhanced macrophage production of the pro-inflammatory cytokines Il-1α, Tnfα, and Il-6. Moreover, oxLDL and its related oxidized lipid moieties promote M1 polarization (Wiesner et al., 2010; Van Tits et al., 2011; Qin et al., 2014) and induce a unique Mox activation status characterized by upregulation of *Hmox1* and *Txnrd1* (Kadl et al., 2010). Here, we found that *PirB* ^MΦKO^ promoted Mox activation upon oxLDL exposure. Taken together, PirB deficiency skews macrophages toward a pro-atherogenic state characterized by enhanced pro-inflammatory M1 polarization as well as Mox activation.

We employed the femoral cuff-induced model of PAD on *PirB* ^MΦKO^ and *PirB* ^flox^ mice to induce peripheral atherosclerosis *in vivo*. Post-mortem histological evaluations of femoral artery sections by Oil Red O staining indicated increased femoral atherosclerotic burden in *PirB* ^MΦKO^ mice, indicating that monocyte/macrophage PirB deficiency enhances peripheral atherogenesis *in vivo*. *PirB* ^MΦKO^ mice also displayed increased levels of plaque macrophage burden. We determined that enhanced macrophage adhesion/rolling on the endothelial luminal surface (as opposed to changes in intra-lesion macrophage proliferation or apoptosis) was primarily responsible for this phenomenon. Consistently, PirB deficiency has been previously shown to produce excessive macrophage adhesion from enhanced integrin signaling (Pereira et al., 2004).

Medial SMCs are responsible for producing the majority of ACTA^+^ SMCs within plaque-stabilizing fibrous caps as well as secreting plaque-stabilizing extracellular matrix components, such as collagen (Misra and Fisher, 2021). Medial SMCs retain their proliferative capacity within plaques, and cues within the plaque microenvironment can regulate plaque SMC proliferation and clonal expansion (Liu and Gomez, 2019). Notably, myeloid-derived plaque macrophages have been shown to inhibit plaque SMC polyclonality (Liu and Gomez, 2019). Consistent with this model, *PirB* ^MΦKO^ mice also displayed increased levels of macrophage burden, medial elastin breaks, and intra-plaque hemorrhage coupled with decreased SMC content, enhanced thinning of SMC-positive fibrous caps, and reduced collagen content, suggesting that myeloid PirB deficiency enhances plaque vulnerability *in vivo* (Chen et al., 2016). However, we did not analyze the mechanism(s) by which monocyte/macrophage PirB deficiency affects plaque SMC phenotype. Future studies should employ macrophage-SMC co-culture studies with *PirB*-null macrophages to thoroughly investigate this question.

Plaque vulnerability has been associated with enhanced intra-plaque Mmp activity on account of their role in fibrous cap matrix degradation (Seifert et al., 2018). As previous work has shown that PirB knockout can produce increases in Mmp-3, Mmp-9, and Mmp-12 expression (Karo-Atar et al., 2013; Kondo et al., 2013), here we analyzed Mmp expression in *PirB* ^MΦKO^ peritoneal macrophages and femoral plaques from *PirB* ^MΦKO^ mice and found increases in Mmp-1, -2, -8, and -9. Notably, the unstable plaque phenotype in human atherosclerotic lesions has been associated with MMP-1 and -8 collagenolytic activity (Sukhova et al., 1999; Molloy et al., 2004) and MMP-9 gelatinase activity (Peeters et al., 2011).

*PirB* ^MΦKO^ mice also displayed increased levels of intra-lesional inflammation and efferocytosis. These findings appear paradoxical, as efferocytosis and inflammation are typically negatively correlated (Gheibi Hayat et al., 2019). Indeed, *PirB* ^flox^ macrophages displayed enhanced pro-inflammatory M1 activation coupled with repression of efferocytosis activity *in vitro*. However, during early plaque formation *in vivo*, oxLDL accumulation produces intra-lesional inflammation and foam cell formation (Lin et al., 2020). These foam cells undergo apoptosis/necroptosis and express surface “eat-me” molecules (e.g., calreticulin18, PtdSer) that interact with their corresponding receptors on phagocytes (e.g., integrin αvβ5, low-density lipoprotein receptor-related protein 1 [LRP1], MERTK, scavenger receptor B, and transglutaminase 2), resulting in efferocytosis activation (Lin et al., 2020). Therefore, these early plaque dynamics explain how high levels of intra-lesional inflammation and efferocytosis can co-exist together *in vivo*.

## Conclusion

The importance of LILRB2 in human atherosclerosis is increasingly evident with reports linking the LILRB2 ligand ANGPTL2 to cardiovascular disease (Gellen et al., 2016; Tian et al., 2016; Tian et al., 2018). In this study, we identify the murine homologue of LILRB2 -- PirB -- as a key regulator in PAD. We show that macrophage PirB reduces peripheral atherosclerotic burden, stabilizes peripheral plaque composition, and suppresses macrophage accumulation in peripheral lesions. Our findings also demonstrate that macrophage PirB inhibits pro-inflammatory activation, inhibits efferocytosis, and promotes lipid efflux, characteristics critical to suppressing peripheral atherogenesis. Further pre-clinical studies will be needed to ascertain the potential of PirB/LILRB2-based therapeutic strategies against PAD.

## Data Availability

The original contributions presented in the study are included in the article/Supplementary Material; further inquiries can be directed to the corresponding author.

## Abbreviations

Angptl2, angiopoietin-like-protein 2; Abca1, ATP binding cassette subfamily A member 1; Abcg1, ATP binding cassette subfamily G member 1; CLI, critical limb ischemia; DEG, differentially-expressed gene; ECs, endothelial cells; FC, fold-change; GO, gene ontology; GS, gene significance; Hmox1, hemeoxygenase-1; IP, immunoprecipitation; ITIM, immunoreceptor tyrosine-based inhibition motif; IFNγ, interferon-γ; KEGG, Kyoto Encyclopedia of Genes and Genomes; LILRB2, leukocyte immunoglobulin-like receptor B2; LPS, lipopolysaccharide; MHC-I, major histocompatibility class I; Mmp, matrix metalloproteinase; MM, module membership; NFκB, nuclear factor-κB; PAD, Peripheral atherosclerotic disease; PPI, protein-protein interaction; Srb1, scavenger receptor class B member 1; SMCs, smooth muscle cells; Shp1, Src homology 2 domain-containing phosphatase 1; Txnrd1, thioredoxin reductase-1; TOM, topological overlap matrix; WGCNA, weighted gene correlation network analysis; WBC, white blood cell.
