# Supplementary material for: Macrophage Paired Immunoglobulin-Like Receptor B Deficiency Promotes Peripheral Atherosclerosis in Apolipoprotein E–Deficient Mice
Source: Front Cell Dev Biol. 2022 Mar 7;9:783954. doi: 10.3389/fcell.2021.783954 (PMC8936951; doi:10.3389/fcell.2021.783954)
Supplement: Supplementary file 3 [file DataSheet1.docx]

**SUPPLEMENTARY INFORMATION FOR**

**Macrophage Paired Immunoglobulin-Like Receptor B (PirB) Deficiency Promotes Peripheral Atherosclerosis in Apolipoprotein E–Deficient Mice**

**SUPPLEMENTARY METHODS**

**Microarray data**

The gene expression profiles of human peripheral atherosclerotic disease (PAD) arterial plaque samples and healthy control samples were obtained from array-based data available in the Gene Expression Omnibus (GEO) database. Three microarray data sets (GSE24702, GSE37824, and GSE100927) were utilized in our analysis (Table S1, Supplementary File 1). From the GSE24702 dataset, all 290 PAD samples were included as PAD samples. From GSE37824, the 133 placebo PAD samples were included as PAD samples. From GSE100927, the 14 infra-popliteal artery PAD samples and the 26 non-control femoral artery PAD samples were included as PAD samples. From GSE100927, the 23 control femoral artery and control infra-popliteal samples were included as healthy control samples.

**Data preprocessing and differentially-expressed gene (DEG) analysis**

The raw files from the three datasets were pre-processed and normalized using limma or RMA-affylmGUI in R Bioconductor. The normalized probes from the three datasets were filtered and merged together to be used as input in the ComBat R package (CRAN R project) for removing the batch effect or non-biological experimental variations using cross-platform normalization ([Johnson et al., 2007](#_ENREF_11)). Batch effect removal is important here, as the data analyzed are derived from multiple platforms and different patient cohorts ([Engchuan et al., 2016](#_ENREF_6)). DEG analysis between PAD and control cohorts was carried out using the limma R package, with thresholds of log|fold-change (FC)| ≥ 1.5 and adjusted *p*<0.05.

**Functional and pathway enrichment analysis**

In order to reveal the pathways that may be associated with the DEGs, the significantly enriched Gene Ontology (GO) terms in Biological processes (BP), Molecular function (MF), and cellular component (CC) as well as the significantly enriched Kyoto Encyclopedia of Genes and Genomes (KEGG) pathways ([Altermann and Klaenhammer, 2005](#_ENREF_1);[Tweedie et al., 2008](#_ENREF_20)) were identified using the Cluepedia plug-in ([Bindea et al., 2013](#_ENREF_2)). The Cluepedia plug-in provides an in-depth view of affected pathways based on *in silico* data ([Bindea et al., 2013](#_ENREF_2)) using Cytoscape network visualization software ([Kohl et al., 2011](#_ENREF_12)). A *p*<0.05 was considered significant. Moreover, the significantly enriched KEGG pathways were also represented by the Pathview function in R ([Luo and Brouwer, 2013](#_ENREF_16)).

**Weighted gene co-expression network analysis (WGCNA)**

WGCNA is a powerful and well-established method for constructing co-expression networks based on gene expression data and is characterized by the utilization of soft thresholding ([Langfelder and Horvath, 2008](#_ENREF_13)). To identify DEGs (*p*<0.05), the CRAN WGCNA module was employed. A soft thresholding power of 5 utilizing the function pickSoftThreshold was also applied in order to establish gene significance (GS), module membership (MM), and module-trait relationships.

**Protein-protein interaction (PPI) network analysis**

To evaluate the protein-level interactions between DEGs identified above, a PPI network was constructed. The protein-coding genes with consistently significant DEGs were predicted as protein pairs using STRING database ([Franceschini et al., 2012](#_ENREF_7)) and a combined score of 0.7 was set. The Cytoscape MCODE plug-in was used to unravel the significant modules of the PPI network that were visualized using Cytoscape software ([Kohl et al., 2011](#_ENREF_12)).

**Kits and reagents**

Custom ELISA detection kits for murine PirB, Il-1α, Il-6, Mcp-1, and Tnfα were obtained from Ray Biotech (Norcross, GA). The Cholesterol assay kit and Apolipoprotein AI kit were obtained from Cell Biolabs (San Diego, CA) and Sigma-Aldrich (St. Louis, MO), respectively. Recombinant mouse interferon γ (IFN-γ) and mouse interleukin 4 (IL-4) were acquired from BioLegend (San Diego, CA). Human high-density lipoprotein (HDL), low-density lipoprotein (LDL), acetylated LDL, and oxidized LDL (oxLDL) were obtained from Yiyuan Biotechnology (Guangzhou, China). The primary antibodies were sourced as follows: anti-α-smooth muscle actin (anti-α-SMA) (ab5694, Abcam), anti-Mac3 (553322, BD Pharmingen), anti-Abca1 (NB400-105), anti-Abcg1 (NB400-132), anti-Srb1 (NB400-104), anti-Mmp-1 (NBP2-22123), Mmp-2 (NB200-193, all Novus Biologicals), Mmp-8 (sc-514803, Santa Cruz Biotechnology), Mmp-9 (NBP2-13173), Mmp-12 (NBP2-67344), Mmp-13 (NBP2-45887), and Mmp-14 (NBP2-67415, all Novus Biologicals).

**Animals**

We created *PirB*^flox/flox^;*Apoe*^−/−^ mice on a C57Bl/6 genetic background (hereinafter *PirB*^flox^ mice) by crossing C57Bl/6 *Apoe*^−/−^ mice (Jackson Laboratory) with C57Bl/6 *PirB*^flox/flox^ mice (Stanford University, Stanford, CA) ([Bochner et al., 2014](#_ENREF_3)) . We further created myeloid-specific *PirB*-null *Apoe*^−/−^ mice (*Lyz*^Cre/+^;*PirB*^flox/flox^;*Apoe*^−/−^) mice on a C57Bl/6 background, hereinafter termed *PirB*^MΦKO^ mice, by crossbreeding C57Bl/6 background *Lyz*^Cre/+^ mice (Jackson Laboratory, B6.129P2-*Lyz2*^tm1(cre)Ifo^/J) with *PirB*^flox^ mice.

**Cuff-induced PAD model**

When the mice were eight weeks old, they were fed a high-fat diet (HFD, also termed Western diet; 42% total kcal from fat, 0.15% cholesterol, Harlan, Indianapolis, IN) for eight weeks. After six weeks on the HFD, mice were subjected to femoral arterial cuff placement to induce peripheral atherosclerosis ([Lardenoye et al., 2000](#_ENREF_14)). Briefly, mice were anesthetized with intraperitoneal (i.p.) 5 mg/kg midazolam, 0.5 mg/kg medetomidine, and 0.05 mg/kg fentanyl. The right femoral artery was exposed and sheathed with a Portex polyethylene cuff (outer diameter 0.80 mm, inner diameter 0.40 mm, and length 2.0 mm). After the procedure, anesthesia was antagonized with atipamezol (1.7 mg/kg) and fluminasenil (0.3 mg/kg), and buprenorphine (0.1 mg/kg) was dosed for pain relief.

Mice were sacrificed two weeks following cuff placement following anesthesia with i.p. midazolam (8 mg/kg), dexdomiter (0.8 mg/kg), fentanyl (0.08 mg/kg), and NaCl (0.9%). Orbital blood was obtained for analysis and stored at −20°C. Left ventricular cardiac puncture was used to apply mild pressure-perfusion (100 mm Hg) with ice-cold PBS for 10 min.

**Oil Red O staining analysis of cuffed femoral arteries**

Following perfusion, the cuffed femoral arteries were harvested. Oil Red O-positive surface areas of e*n face* preparations of whole femoral arteries was used to quantify overall femoral arterial atherosclerotic burden as previously described ([Sukhanov et al., 2007](#_ENREF_18)).

**Histopathological analysis of femoral atherosclerotic lesion sections**

All histopathological analyses were performed by investigators blinded to the sample’s group assignment. Whole femoral arteries were fixed overnight in 4% formaldehyde in PBS and paraffin-embedded with an automated tissue processor (Leica, Germany). Histopathological analysis was performed by blinded investigators who were provided with anonymized fixed whole femoral arteries. Twenty cross-section samples (5 µm each), separated by 60-μm intervals, were cut and excised from the whole length of the paraffin-embedded femoral artery. The cross-section samples were examined by histological staining using Carstairs’ method. This method detects RBCs (yellow-red), smooth muscle cells (SMCs) (dark red), collagen (bright blue) and fibrin (orange-red) ([Gough et al., 2006](#_ENREF_8)).

For measuring macrophage-positive lesion areas, xylene-deparaffinized cross-section samples were stained using an anti-mouse Mac3 antibody (553322, BD Pharmingen) and an anti-mouse α-SMA antibody (ab5694, Abcam). Additionally, the cross-section samples were stained for Masson’s Trichome (Richard-Allan Scientific, Kalamazoo, MI). Atherosclerotic plaque cross-sections were localized based on the foregoing macrophage-positive lesion analysis. Plaque cross-sectional areas were measured by H&E staining of cross-section samples using standardized protocols ([Sukhanov et al., 2007](#_ENREF_18)).

To analyze macrophage apoptosis and efferocytosis *in situ*, we applied a previously described protocol ([Doran et al., 2017](#_ENREF_5)). Briefly, xylene-deparaffinized cross-section samples were stained with TUNEL (Roche), anti-mouse Mac3 antibody (553322, BD Pharmingen), or anti-mouse Mertk (ab95925, Abcam) as indicated. Apoptotic cells were counted as either “macrophage-associated” (i.e., co-localization with Mac3 staining) or “free” (i.e., no co-localization with Mac3 staining). Efferocytosis levels were assessed by the ratio of macrophage-associated TUNEL^+^ (Mac3^+^TUNEL^+^) cells-to-free TUNEL^+^ cells ([Yurdagul Jr et al., 2020](#_ENREF_23)).

To identify plaque vulnerability, features were assessed using criteria proposed by Gough et al ([Gough et al., 2006](#_ENREF_8)). Fibrous cap disruption was considered when at least three cross-section samples (separated by 60-μm intervals) demonstrated a disruption or discontinuity in the elastin layer of the cap, whereas a break was scored for an animal showing a clear fibrous cap break within the lumen. Presence of RBCs in at least three cross-section samples (separated by 60-μm intervals) was scored as intraplaque hemorrhage, and any discontinuous lamellae of the median elastin wall were scored for median elastin breaks. At least three cross-section samples (separated by 60-μm intervals) showing the presence of orange-red Carstair’s staining and additionally staining positive by anti-fibrin/fibrinogen immunostaining was defined to be positive for fibrin deposition.

**Blood cell count**

Peripheral blood smears were used to count the total white blood cells. The monocyte numbers were calculated following protocols described elsewhere ([Wang et al., 2012](#_ENREF_22)). Briefly, whole blood cells and a cocktail of monoclonal antibodies against B-cells (B220-PE, clone RA3-6B2, ThermoFisher), T-cells (CD90-PE, clone 53-2.1, ThermoFisher), NK cells (CD49b-PE, clone DX5; NK1.1-PE, clone PK136, ThermoFisher), myeloid cells (CD11b-APC, clone M1/70, ThermoFisher), granulocytes (Ly6G–PE, clone 1A8-Ly6G, ThermoFisher) and monocyte subsets (Ly6C–FITC, clone AL-21, BD Pharmingen) were mixed and incubated together. Using a standard flow cytometer (Beckman-Coulter), circulating monocytes were identified as CD11b^hi^/CD90^lo^/B220^lo^/CD49b^lo^/NK1.1^lo^/Ly6G^lo^ cells. To calculate monocyte numbers, the total leukocyte counts (as obtained in the blood smears) were multiplied by the percentage of cells within the monocyte gate in the fraction of mononuclear cells. Within this population, Ly-6C^hi^ inflammatory monocyte subsets were also identified.

**Tracing recruitment of circulating monocytes**

For labeling Ly6C^hi^ inflammatory monocytes, we used methods adapted from those described elsewhere ([Tacke et al., 2007](#_ENREF_19)). 24 h prior to injecting 250 μl of 1X PBS diluted (1:25) 0.5-μm Fluoresbrite® Polychromatic Red Microspheres (2.5% solids in water, Polysciences Inc.) into the Western diet-fed mice lateral tail veins 3 and 7 days prior to their sacrifice, 200 μl of liposome encapsulated dichloromethylene-bisphosphonate (clodronate) (Sigma-Aldrich) or vehicle control liposomes were injected intravenously. Clodronate liposome injections deplete monocytes from the circulation. The femoral artery serial sections obtained were stained with Oil Red O for visualizing lesion area, anti-mouse Mac3 antibody (553322, BD Pharmingen), or DAPI. Red microsphere-labeled cells were also Mac3-immunopositive, confirming labeling specificity. The number of red microsphere-positive cells were counted in a plaque and normalized to the efficiency of labeling in circulating monocytes (% labeled/total number) and assessed by flow cytometry.

**Intravital fluorescence microscopy**

Intravital imaging was carried out by fluorescence microscopy using methods adapted from those described elsewhere ([Wang et al., 2013](#_ENREF_21)). Briefly, 1 h before the assessment, anti-CD11b Alexa Fluor 488 antibody (clone M1/70, ABLab, Vancouver, B.C., Canada) and anti-Ly6C Alexa Fluor 700 antibody (clone AL-21, BD Pharmingen) were administered intravenously in mice *via* the jugular vein. Mice were then positioned supine position on a Plexiglas microscope stage and a small section of mesentery from the small intestine was exteriorized on a glass slip and prepared for microscopic observation using an inverted microscope (Eclips TE2000, Nikon), by super fusing with warm bicarbonate-buffered saline (37°C, pH 7.4). Using a thermostat regulated heat lamp, the body temperature of the mice was retained between 36.5°C and 37.5°C. Using the CoolSNAP ES high-resolution charge-coupled device (CCD) camera from Photometrics, fluorescent images were captured. We observed nearly 10 single un-branched venules from the intestinal segment of about 20–50 μm in diameter and 100 μm lengths, for at least 1 min.

To access the interaction between CD11b^+^Ly6C^+^ inflammatory monocytes and endothelia, we quantified the number of rolling and firmly attached monocytes in each venule and the mean of 10 un-branched venules were considered for analysis. If the CD11b^+^Ly6C^+^ monocytes did not show any movement for 30 s (normalized in terms of the number of adherent monocytes per square millimeter of endothelial surface), they were considered adherent and for rolling monocyte, those crossing a cross line at a velocity significantly slower than the centerline velocity were counted and the CD11b^+^Ly6C^+^ monocyte rolling flux was expressed as monocytes per minute.

**Laser capture microdissection (LCM) of macrophage-rich plaque areas**

Femoral artery cross-sections were cut and placed on PEN membrane glass slides. Using Histogene® LCM Frozen Section Staining Kit (Thermo Fisher), the slides were processed instantly. Further, the slides were fixed with 75% ethanol and stained with Histogene staining solution. The rat anti-mouse macrophage marker antibody (sc-101447, Santa Cruz Biotechnology) was used to stain the serial sections, and these were developed using anti-rat Alexa488 secondary antibody (Invitrogen) plus DAPI. LCM was performed with the Applied Biosystems® ArcturusXT™ LCM System by using a Nikon Eclipse Ti-E inverted research microscope and a combination of IR and UV lasers to provide laser-capture and laser cutting into one modular platform. RNA isolation was performed from LCM-dissected tissue kept on dry ice using Arcturus® PicoPure® Frozen RNA Isolation Kit (Thermo Fisher). The quality and concentration of RNA were estimated using Take3 plates on Cytation5 imager (Bio-Tek). Eight ng of such high-quality RNA was used to perform cDNA synthesis using Arcturus® RiboAmp® HS Plus cDNA kit (ThermoFisher) according to the manufacturer’s instructions. Real-time quantitative PCR (qPCR) was performed as described below.

**Macrophage culture**

Peritoneal cells from *PirB*^MΦKO^ and *PirB*^flox^ mice were thioglycollate-elicited to obtain macrophages using methods adapted from those described elsewhere ([Zhang et al., 2008](#_ENREF_24)). Briefly, mice were injected intraperitoneally (IP) with sterile thioglycolate and four days later peritoneal cells were collected using peritoneal lavage. Cells recovered were cultured overnight in RPMI 1640 medium containing 10% FBS. Cells that did not adhere were washed with 1X PBS, supplemented with fresh media and were further grown overnight. The purity of the macrophages grown was confirmed using anti-F4/80 antibodies and found to be >95%. These cells were polarized to M1 macrophages by exposing to IFNγ for 6 hours at 150 U/mL followed by exposure to LPS for 18 hours at 50 ng/mL and were further induced to M2 macrophages by exposing to IL4 for 24 hours at 20 U/mL. To test the effects of oxLDL, which contributes to the atherosclerotic plaque formation and progression, macrophages were initially primed for 6 hours with IFNγ, followed by 18 hours of oxLDL exposure at 50 μg/mL.

**Wild-type and mutant *PirB* gene overexpression *in vitro***

The murine *PirB* ORF clone (NM_011095, cat#: MC221998, Origene) was amplified with a PrimeSTAR PCR mix (Takara, Japan). As previously described ([Cui et al., 2020](#_ENREF_4)), the purified PCR product was then subcloned into a pcDNA3.1(-) vector to form the wild-type (WT) PirB overexpression vector. Site-directed mutagenesis, in which the 3 ITIM tyrosine residues were mutated into phenylalanine by three rounds of point mutant PCR ([Lu et al., 2018](#_ENREF_15)), was applied to this PirB-pcDNA3.1(-) to create the PirB^3Y-F^ mutant. This PirB^3Y-F^ mutant sequence was then subcloned into a pcDNA3.1/myc-His(-) C vector. Cells (2×10^6^) were transfected with the PirB-pcDNA3.1(-) vector (2 μg) or the PirB^3Y-F^-pcDNA3.1/myc-His(-) C vector (2 μg) by electroporation using an Amaxa 4D-Nucleofector (Lonza). Transfection efficacy was assessed by immunoblotting.

***In vitro* apoptotic cell uptake and clearance**

As previously described ([Heo et al., 2014](#_ENREF_9)), thymocytes isolated from WT C57Bl/6 mice were cultured overnight in RPMI 1640 containing 10% FBS, and apoptotic thymocytes were created by exposure to 100 nM dexamethasone for three hours. Then, the apoptotic thymocytes were labeled with CellTrackerTM Green CMFDA-FITC (#C7025, Molecular Probe). For uptake experiments, we added peritoneal macrophages derived from *PirB*^MΦKO^ and *PirB*^flox^ mice to apoptotic thymocytes at a 2.5:1 thymocyte:macrophage ratio and co-cultured them for 60 minutes. Double-discrimination flow cytometry was used to distinguish internalized from externally-bound apoptotic thymocytes in order to determine the CMFDA+ macrophage percentage. For double-feeding experiments, peritoneal macrophages derived from *PirB*^MΦKO^ and *PirB*^flox^ mice were incubated with apoptotic thymocytes at a 2.5:1 thymocyte:macrophage ratio for 24 h and then re-challenged with CMFDA-labeled apoptotic thymocytes. One day thereafter, the phagocytic index was calculated by fluorescent microscopy imaging as previously described ([Heo et al., 2014](#_ENREF_9)). In brief, 200 cells per sample were counted; the following formula was used: [(no. of macrophages containing one apoptotic thymocyte (AT)) + 2 × (no. of macrophages containing two ATs) + 3 × (no. of macrophages containing three ATs) + 4 × (no. of macrophages containing more than four ATs)] / total no. of macrophages].

**Immunoprecipitation (IP) and Western blotting analysis**

IP was carried out using standard procedures described previously. At 24 hours following transfection, cells were lysed in an ice-cold IP buffer (150 mmol/L NaCl, 50 mmol/L Tris-HCl, 5 mmol/L EDTA, 5% glycerol, 1% Triton X-100, and a Roche protease inhibitor cocktail). The lysates were then incubated with anti-Traf6-conjugated agarose beads (sc-8409, SCBT), anti-Jak1-conjugated agarose beads (sc-1677, SCBT), or normal mouse IgG-conjugated agarose beads (sc-2343, SCBT) at 4°C under rotation for 4 hours. The agarose beads were washed three times with cold PBS and eluted. Western blotting was then performed as described below.

Western blotting was carried out using standard procedures described previously ([Higashi et al., 2008](#_ENREF_10)). Briefly, the cells were lysed with RIPA buffer (150 mmol/L NaCl, 0.1 mol/L okadaic acid, 1 mmol/L EDTA, 20 mmol/L Tris-HCl, 5 mmol/L dithiothreitol, pH 7.2, 0.1 mmol/L phenylmethylsulfonyl fluoride, 1 mmol/L sodium orthovanadate, 0.1 mol/L aprotinin, 1% Nonidet P-40, 10 g/mL leupeptin, and 10 mmol/L NaF), and protein estimation in the lysate were carried out. Nearly 15–20 mg of total protein was run on 10% SDS-PAGE, which was followed by immune blotting analysis using primary antibodies described earlier and HRP-tagged secondary antibodies. Enhanced chemiluminescence (Amersham) was used to detect immunopositive bands. Blots were stripped of the primary antibodies, washed with 1× PBS, blocked and again re-probed with monoclonal anti-β-actin antibody, which was used as a loading control.

**Real-time quantitative PCR (qPCR)**

Total RNA was extracted, and qPCR was performed following protocols described elsewhere ([Sukhanov et al., 2006](#_ENREF_17)). Briefly, total RNA from femoral artery tissue was extracted using TriPure Isolation Reagent (Roche) and was further purified using the RNeasy mini kit (Qiagen). First Strand cDNA Synthesis kit (Amersham) was used to reverse-transcribe the mRNA into cDNA. qPCR was performed with primers from Qiagen (RT² qPCR Primer Assay) using a 2-step, 40-cycle program, in the iCycler IQ Real-Time Detection System (Bio-Rad). The housekeeping gene *Actb* was used as the housekeeping control. Their relative expression was then calculated using the comparative Ct method (ΔΔCT).

**LXRE luciferase reporter assay**

The TK-LXREx3-Luc LXRE-luciferase vector was kindly gifted by the Mangelsdorf lab at the University of Texas (UT) Southwestern Medical Center. Macrophages were co-transfected with this TK-LXREx3-Luc vector and phRL-TK Renilla luciferase vector with X-tremeGENE 9 DNA transfection reagent (Roche). Following 24 h of incubation, firefly and Renilla luciferase activities were assayed using a dual-luciferase reporter assay (Promega) on a microplate reader (BioTek).

**Statistical analysis**

GraphPad PRISM (version 6.07) software was used to perform the statistical analyses. Data are expressed as means ± standard deviations (SDs). Residual distribution of the data was evaluated by D’Agostino-Pearson omnibus normality test and equality in variances assessed using Levene’s test. In accordance with the normality of residuals distribution, differences in outcomes were resolved using Mann-Whitney *U*-test, unpaired Student’s *t*-test, or one-way or two-way analysis of variance (ANOVA) with Bonferroni’s post-hoc test as indicated in the Figure Legends. *P*<0.05 was considered significant for all analyses. Fisher’s exact test was used to compare the frequency of observed indices of plaque vulnerability.

**SUPPLEMENTARY TABLES**

**Table S1. Characteristics of the GEO Datasets**

| **GEO ID** | **Platform ID** | **PAD samples** | **Control samples** |
| --- | --- | --- | --- |
| GSE100927  *Atherosclerotic and control peripheral artery gene expression* | GPL17077  Agilent-039494 SurePrint G3 Human GE v2 8x60K Microarray 039381 (Probe Name version) | Non-control femoral artery (n=26)  Infra-popliteal artery (n=14) | Femoral artery controls (n=12)  Infra-popliteal artery controls (n=11) |
| GSE37824  *Evaluation of a novel clinical platform for cardiovascular drug development* | GPL4372  Rosetta/Merck Human 44k 1.1 microarray | All placebo-treated samples (n=132) | None |
| GSE24702  *Gene expression profiling of human atherosclerotic plaque: 290 peripheral plaques* | GPL10687  Rosetta/Merck Human RSTA Affymetrix 1.0 microarray, Custom CDF | All samples (n=290) | None |
| Totals |  | n=462 | n=23 |

**Table S2. PirB^MΦKO^ does not impact circulating monocyte counts or activation**

|  | **PirB-flox (mean ± SD)** | | | **PirB^MΦKO^ (mean ± SD)** | | | | ***P*-values by two-way ANOVA** |
| --- | --- | --- | --- | --- | --- | --- | --- | --- |
|  | **Regular chow (n=12)** | | **HFD (n=18)** | **Regular chow (n=12)** | | **HFD (n=18)** | |  |
| Total monocytes  (per mm^3^) | 57.00 ± 3.22 | | 227.44 ± 58.08 | 62.42 ± 2.78 | | 237.67 ± 53.50 | | Diet:  *p*<0.0001**;  PirB^MΦKO^:  *p*=0.47 (NS) |
|  |  |  |  |  |  |  |  |  |
| Ly6C^hi^ monocytes  (% of total monocytes) | 64.08 ± 8.24 | | 72.00 ± 7.87 | 68.08 ± 7.89 | | 74.56 ± 8.12 | | Diet:  *p*= 0.0012**;  PirB^MΦKO^:  *p*=0.14 (NS) |

**p*<0.05, ***p*<0.01; NS, non-significant

**Table S3. HFD-fed PirB^flox^ and PirB^MΦKO^ mice show no differences in circulating cholesterol and pro-inflammatory cytokine levels**

|  | **HFD PirB^flox^ (n=18)** | | **HFD PirB^MΦKO^ (n=18)** | | ***P*-values by *t*-test** |
| --- | --- | --- | --- | --- | --- |
|  | **Mean** | **± SD** | **Mean** | **± SD** |  |
| Cholesterol (mg/dL) | 3329.9 | ± 177.2 | 3254.5 | ± 126.8 | 0.15 (NS) |
| TNFα (pg/mL) | 7.4 | ± 1.7 | 7.6 | ± 1.7 | 0.72 (NS) |
| IL-6 (pg/mL) | 8.4 | ± 2.6 | 8.6 | ± 2.7 | 0.82 (NS) |
| MCP-1 (pg/mL) | 158.4 | ± 52.4 | 156.3 | ± 19.0 | 0.87 (NS) |

**p*<0.05, ***p*<0.01; NS, non-significant

**Table S4. PirB^MΦKO^ induces femoral artery plaque vulnerability in HFD mice**

| **Plaque characteristic** | **HFD PirB^flox^** | **HFD PirB^MΦKO^** | ***P*-values by *t*-test** |
| --- | --- | --- | --- |
| Fibrous cap disruption | 0/18 | 1/18 | 0.32 (NS) |
| Intraplaque hemorrhage | 2/18 | 11/18 | 0.0021** |
| Fibrin deposition | 0/18 | 0/18 | NS |
| Medial elastin breaks | 0/18 | 6/18 | 0.0082** |

**p*<0.05, ***p*<0.01; NS, non-significant

**SUPPLEMENTARY FIGURES**

**Figure S1.** **Significant differentially-expressed genes (DEGs)**

The volcano plot depicts the DEGs between healthy and PAD samples.

**Figure S2. The significantly enriched pathways for differentially-expressed genes (DEGs)**

**(A)** List of GO terms and KEGG pathways as assessed using Cluepedia. **(B)** GO terms and KEGG pathways represented as a network.

**Figure S3. Module construction using weighted gene correlation network analysis (WGCNA)**

**(A)** Determination of the optimum amount of soft power needed to generate the adjacency matrix and topological overlap matrix (TOM). **(B)** Heatmap depicting the TOM supplemented by hierarchical-clustering dendrograms and module colors. **(C)** Dendrogram showing gene module identification by hierarchical clustering based on the TOM dissimilarity measure. Dendrogram (top panel) represents the clustering of genes in a hierarchy, with all gene modules (bottom panel) shown with different color codes. **(D)** Following merging of small modules, dendrogram (top panel) showing the 22 gene modules (bottom panel) with a satisfactory soft power cut-off of 5 in PAD condition.

**Figure S4. Microarray expression data for the WGCNA modules**

Microarray showing gene expression patterns for the 22 WGCNA-derived modules.

**Figure S5. WGCNA module correlation plot**

This plot quantifies the similarity among the 22 WGCNA-derived modules by eigengene correlation.

**Figure S6. Correlation analysis between WGCNA modules and phenotype traits**

For each module, the top number indicates the association between the module and phenotype trait (PAD and control) represented as a correlation coefficient, while the bottom number indicates the significance of this association by *p*-value. The most characteristic modules associated with the PAD trait are the turquoise and pink modules.

**Figure S7. Correlation analysis between WGCNA module membership and gene significance for PAD**

Scatter plots for module membership (MM) and gene significance (GS) for genes in **(A)** turquoise module (*r*=0.49, *p* =1.2e-19) and the (**B**) pink module (*r*=0.48, *p=9.4*e-19).

**Figure S8. Top clusters in the protein-protein interaction (PPI) network**

PPI network analysis reveals four clusters: **(A)** cluster 1 (score = 28.00) consisting of 28 nodes and 378 edges, **(B)** cluster 2 (score = 19.24) consisting of 22 nodes and 202 edges, **(C)** cluster 3 (score = 13.69) consisting of 13 nodes and 89 edges, and **(D)** cluster 4 (score = 13.47) consisting of 16 nodes and 101 edges. Pink circles represent the upregulated genes, and the blue circle represent the downregulated genes.

**Figure S9. PirB knockout and Akt activation in *PirB*^MΦKO^ peritoneal macrophages**

**(A)** PirB protein expression in *PirB*^MΦKO^ and *PirB*^flox^ peritoneal macrophages and neutrophils detected by Western blotting. Thioglycolate-treated peritoneal macrophages isolated 24 h after treatment by centrifugal isolation using Percoll (63%). Flow cytometry by CD11b^hi^/F4/80^hi^ or CD11b^hi^/Ly6G^hi^ markers confirmed the purity of macrophages and neutrophils, respectively. 10 μg macrophage protein and 100 μg neutrophil protein were used in the analysis. **(B)** Peritoneal macrophages exposed to either murine MHC-I β2M (6.25 ng/mL) or murine Angptl2 (0.5 μg/mL) were analyzed for PirB, phospho-Akt (p-Akt^S473^), Akt, and β-actin levels using Western blot analysis. **(C)** Peritoneal macrophages show MHC-I β2M dose-dependent response in Akt phosphorylation. Quantification of p-Akt^S473^ levels in peritoneal macrophages exposed to the indicated doses of MHC-I β2M for 15 minutes. **(D)** PirB knockout abolishes PirB/p-Shp1 interaction. Anti-PirB immunoprecipitates from peritoneal macrophage lysates were resolved by SDS–PAGE followed by Western blot. p-Shp1/Shp1 ratios in the anti-PirB immunoprecipitates were quantified. Equal detection in lanes loaded with the same amounts of lysate confirmed immunoprecipitation efficiency (~100%). All *in vitro* experiments: *n*=3 biological replicates × 3 technical replicates.

**Figure S10.** ***PirB*^MΦKO^ mice show signs of enhanced plaque vulnerability**

Representative femoral artery lesions from **(A)** *PirB*^flox^ and **(B)** *PirB*^MΦKO^ mice stained by Carstair’s stain. Lower collagen deposits in *PirB*^MΦKO^ plaques reflected by thinner blue staining. Plaque vulnerability in *PirB*^MΦKO^ plaques seen by the presence of RBCs within lesions.

**Figure S11. Macrophage *PirB* knockout inhibits alternative M2 macrophage polarization *in vitro***

Adherent *PirB*^flox^ and *PirB*^MΦKO^ peritoneal macrophages were either non-activated (NA) or exposed to IL-4 for M2 activation. qPCR analysis for the M2 activation markers **(A)** *Arg1*, **(B)** *Mrc1*, and **(C)** *Pparg*. **p*<0.05, ***p*<0.01 [two-way ANOVA with Bonferroni post-hoc test]. All *in vitro* experiments: *n*=3 biological replicates × 3 technical replicates.

**Figure S12. Cultured *PirB*^MΦKO^ macrophages display upregulated efferocytosis under oxLDL-treated conditions**

Adherent *PirB*^MΦKO^ and *PirB*^flox^ peritoneal macrophages were either non-activated (NA) or exposed to IFNγ and LPS for M1 activation or IL-4 for M2 activation in the presence or absence of oxLDL for 24 h. **(A-E)** qPCR analysis of the efferocytosis markers **(A)** *Anxa1*, **(B)** *Gas6*, **(C)** *C1qa*, **(D)** *Mertk*, and **(E)** *Mfge8*. **(F)** Apoptotic thymocyte phagocytosis by peritoneal macrophages *in vitro* after 60 minutes of incubation with CMFDA–labeled apoptotic thymocytes at a 2.5:1 apoptotic thymocyte:macrophage ratio. The chart reports the percentages of CMFDA^+^ macrophages by flow cytometry. **(G, H)** Peritoneal macrophages were fed CMFDA-labeled apoptotic thymocytes (**(G)** single feed macrophages) 24 hours before refeeding them with CMFDA-labeled apoptotic thymocytes (**(H)** double feed macrophages) at a 2.5:1 apoptotic thymocyte:macrophage ratio. Macrophages were quantified by flow cytometry 24 hours after each feeding session. Phagocytic indices were calculated as described in Methods. **p*<0.05, ***p*<0.01 [two-way ANOVA with Bonferroni post-hoc test]. All *in vitro* experiments: *n*=3 biological replicates × 3 technical replicates.

**Figure S1**

**
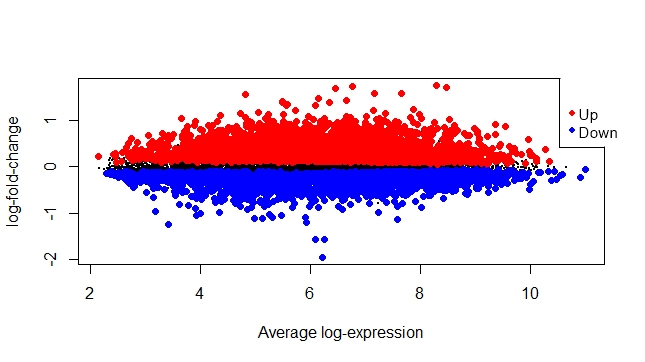
**

**Figure S2**

**
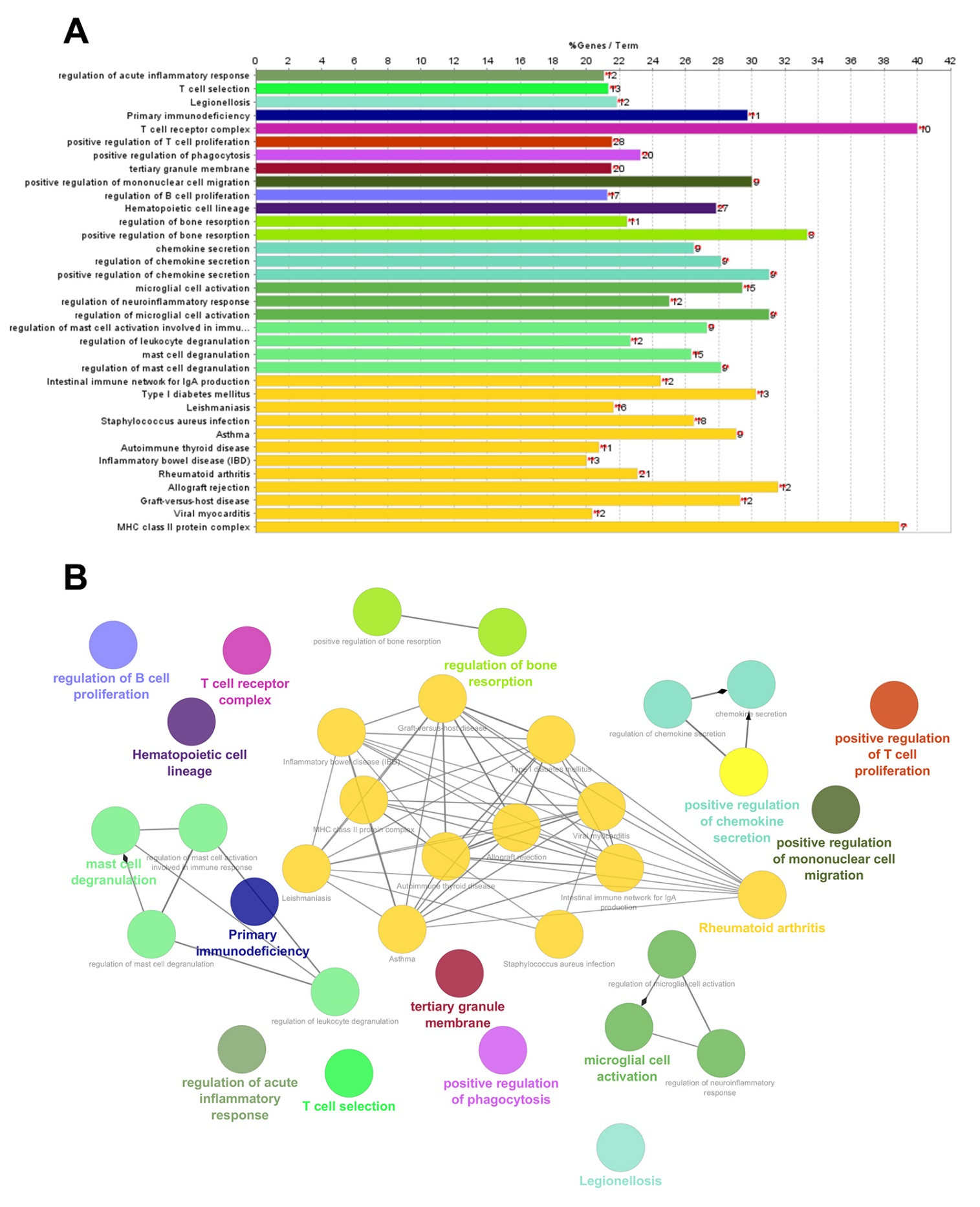
**

**Figure S3**

**
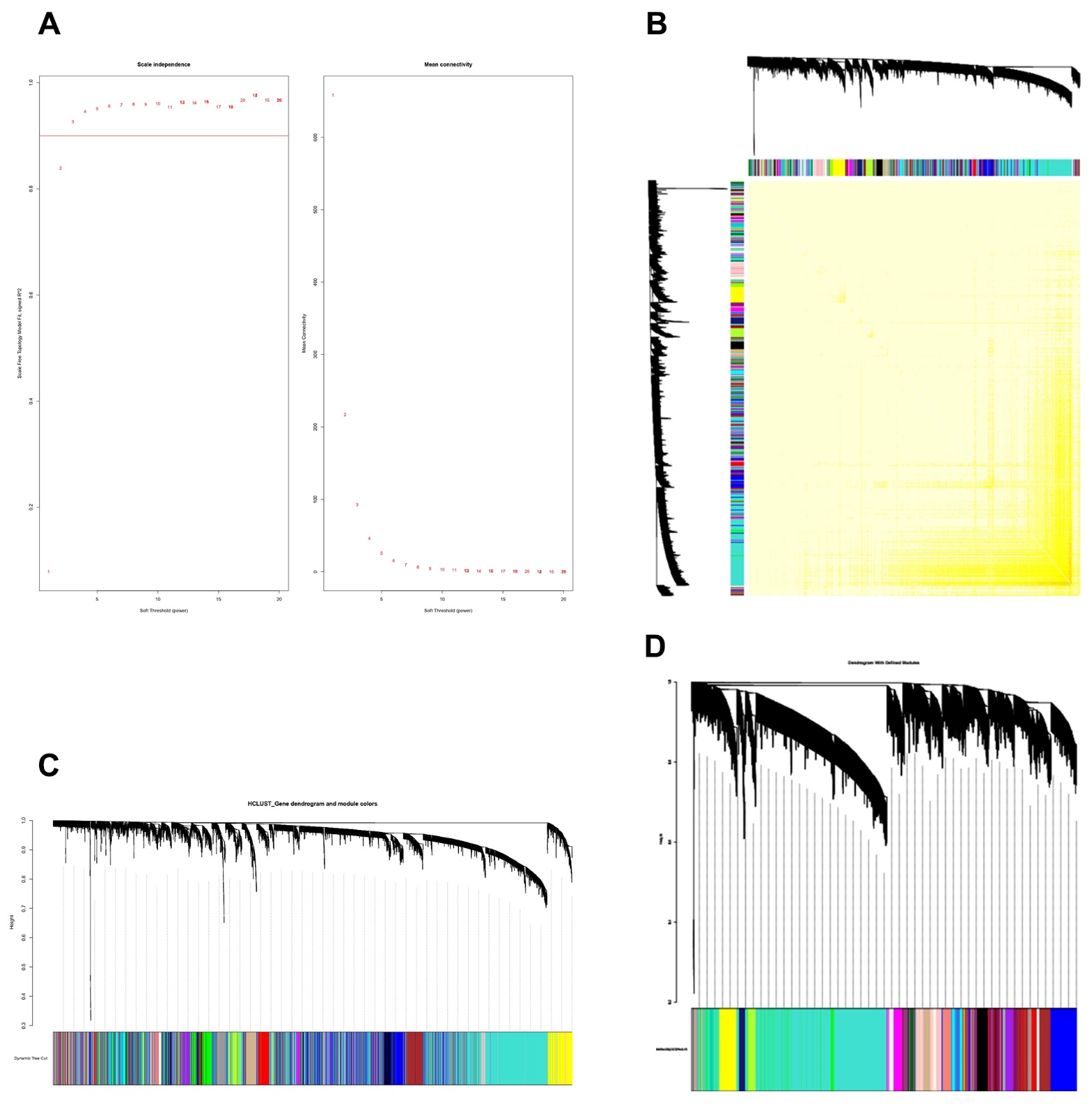
**

**Figure S4**

**
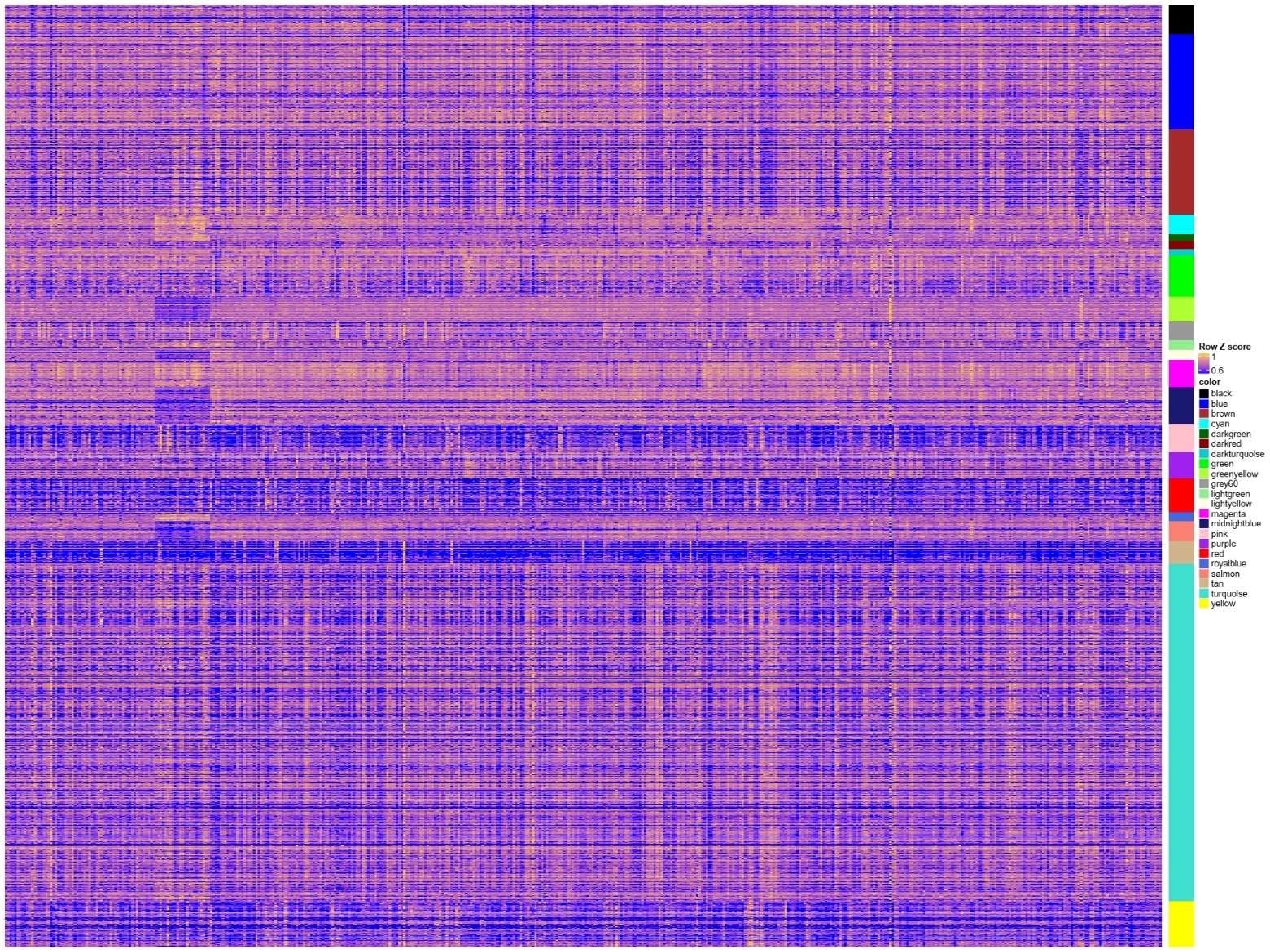
**

**Figure S5**

**
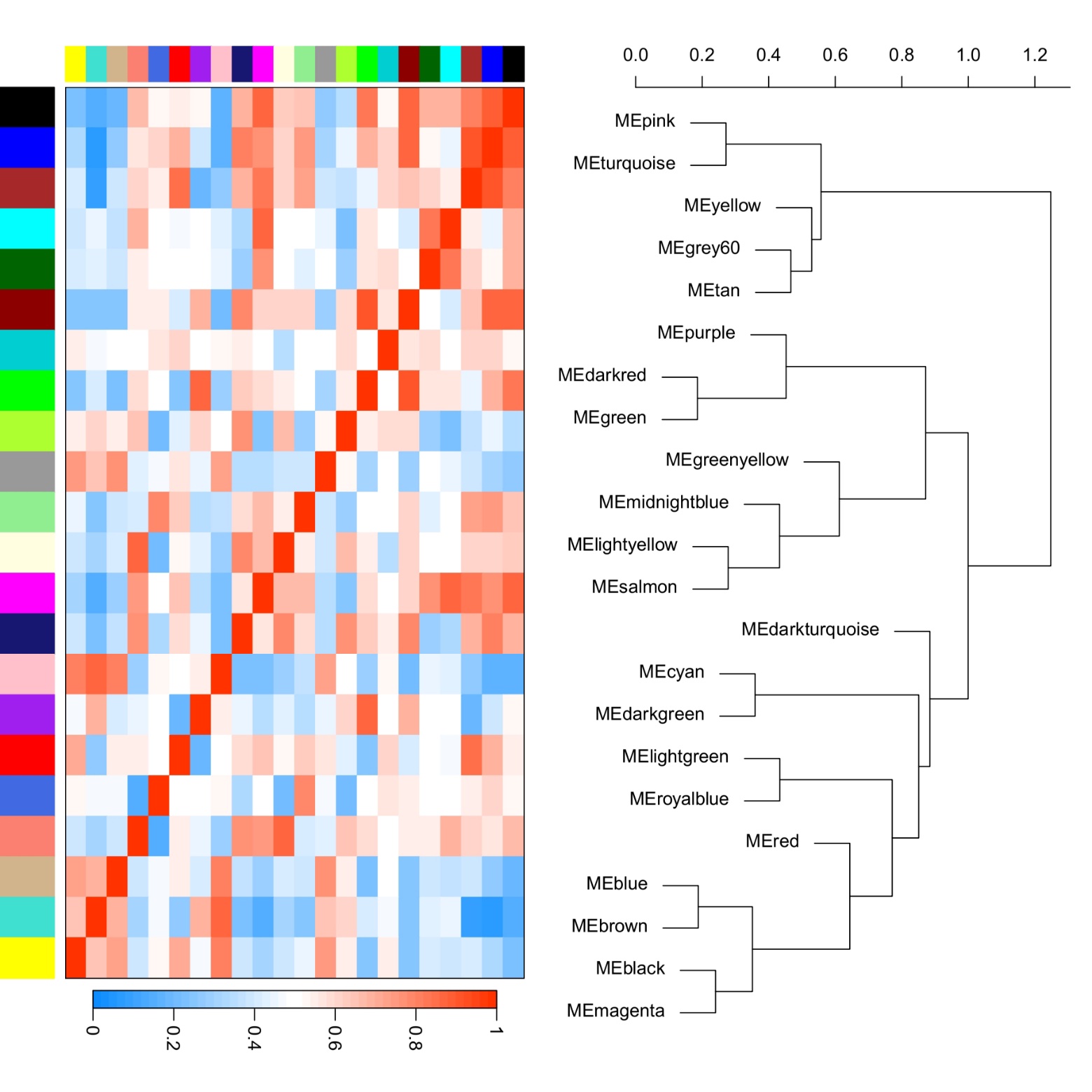
**

**Figure S6**

**
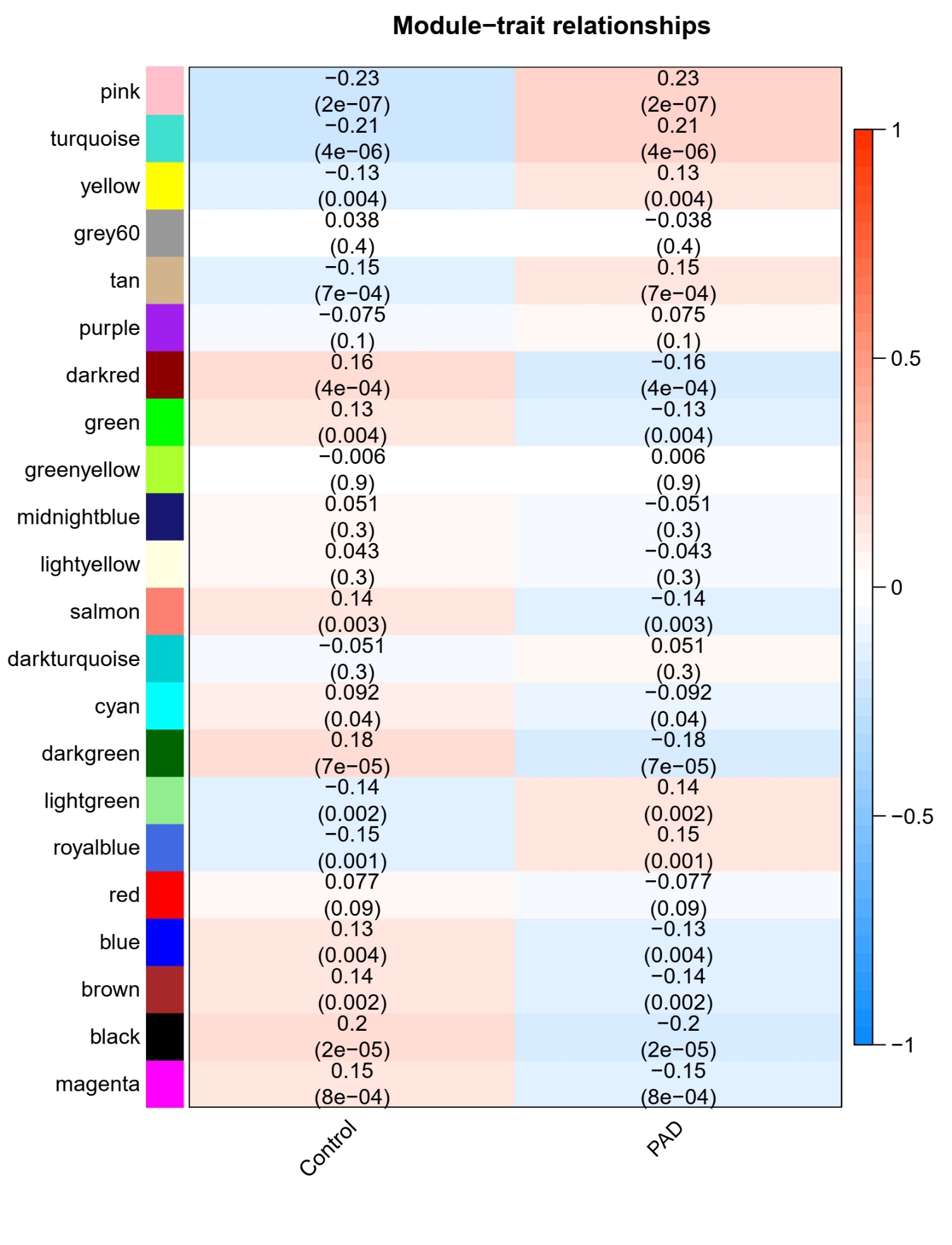
**

**Figure S7**

**
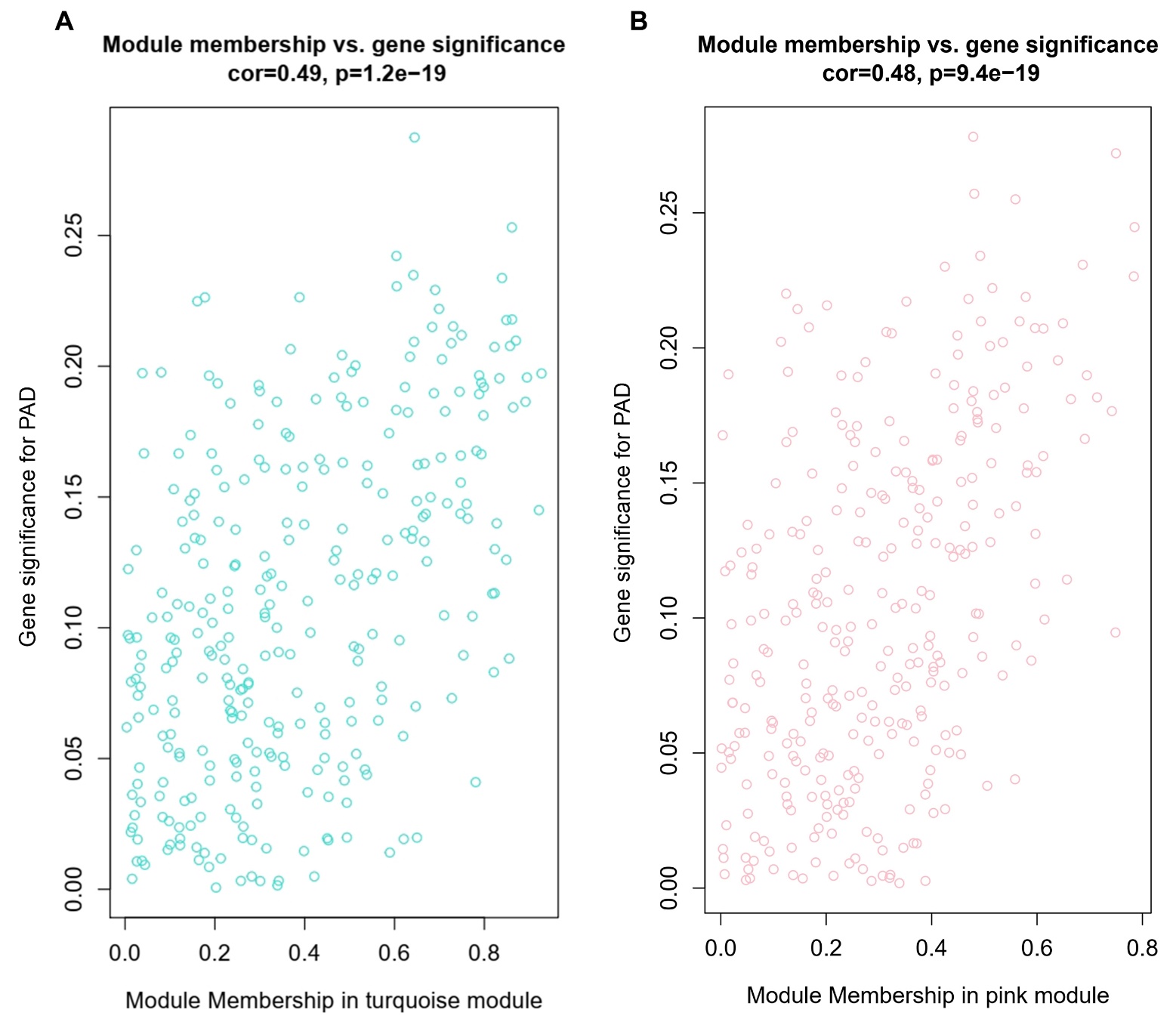
**

**Figure S8**

**
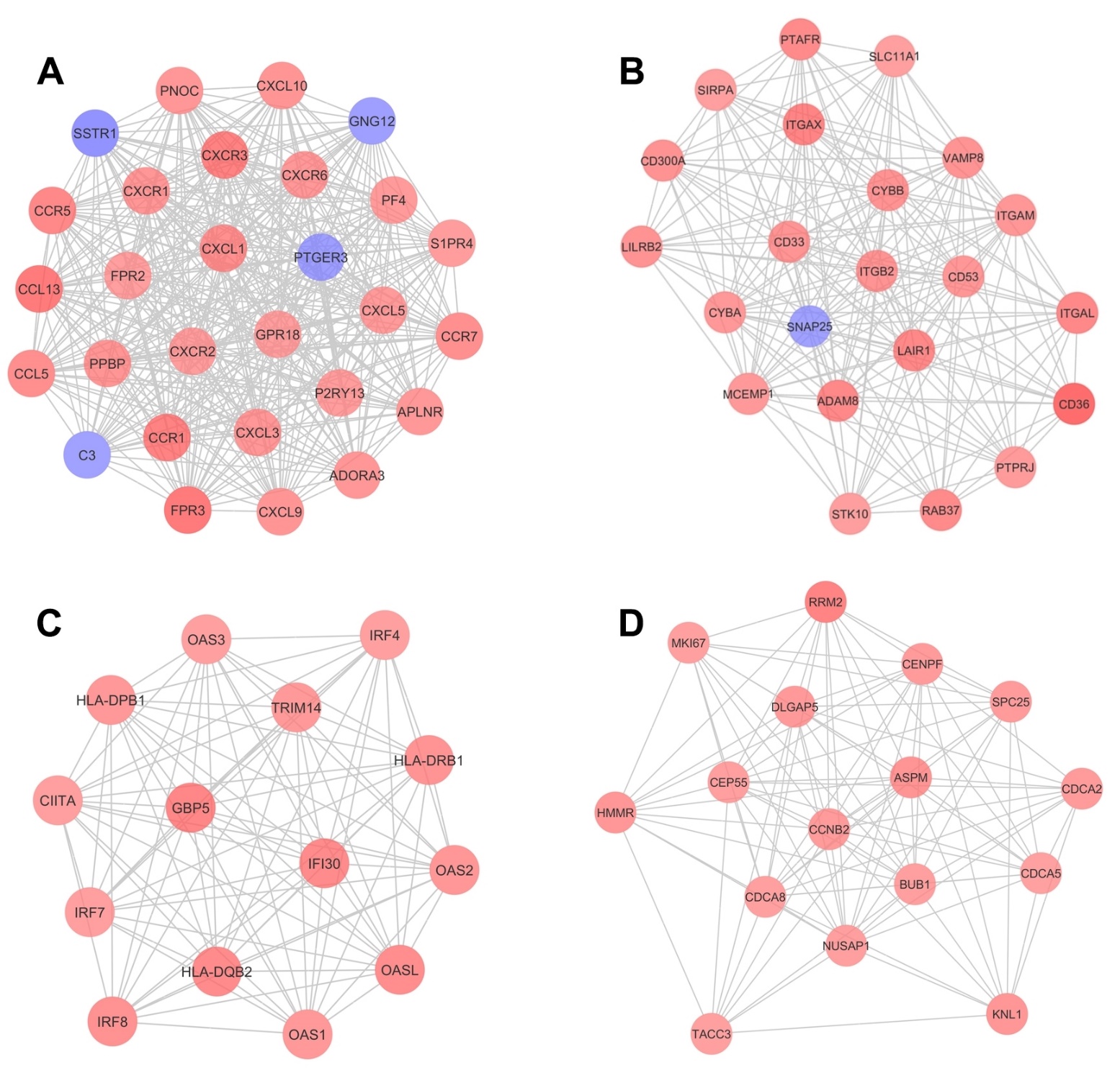
**

**Figure S9**


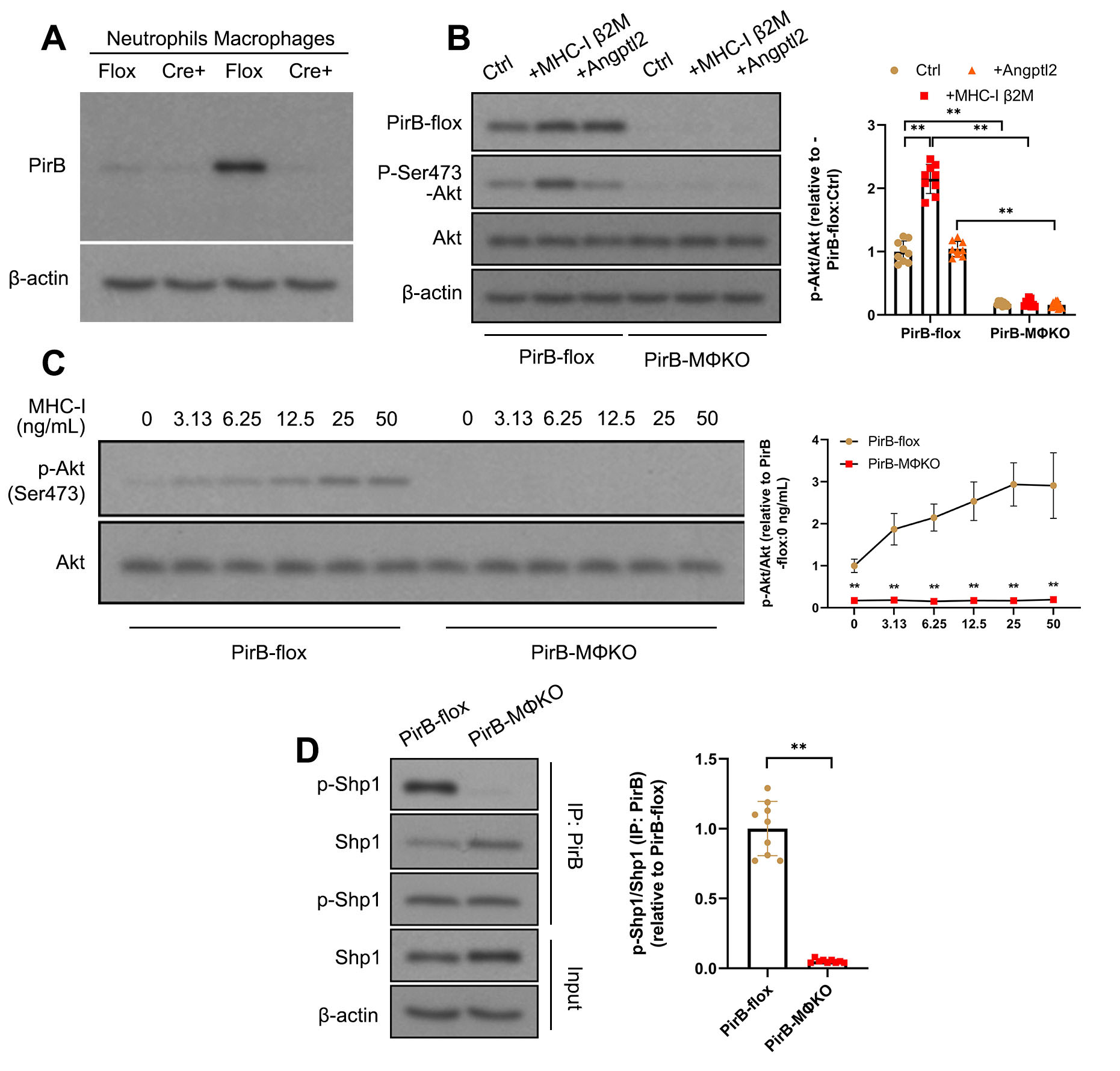


**Figure S10**

**
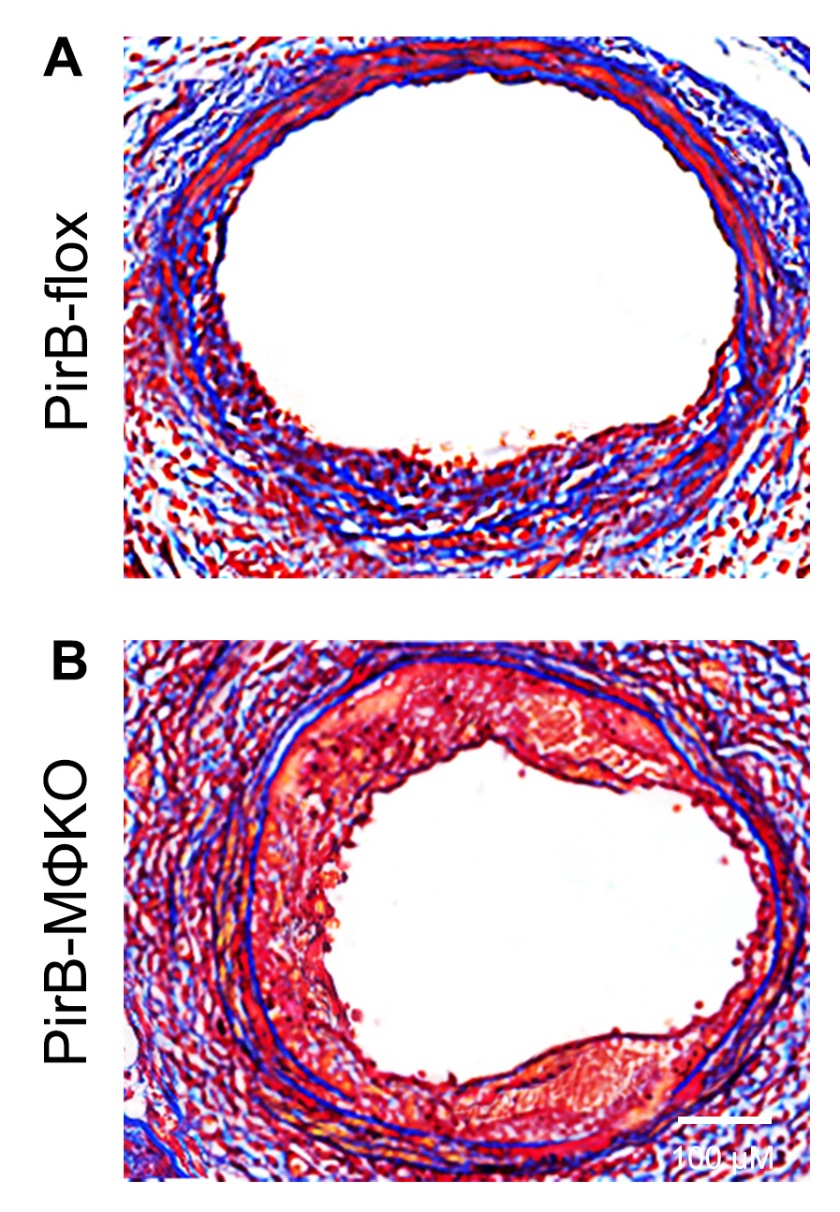
**

**Figure S11**


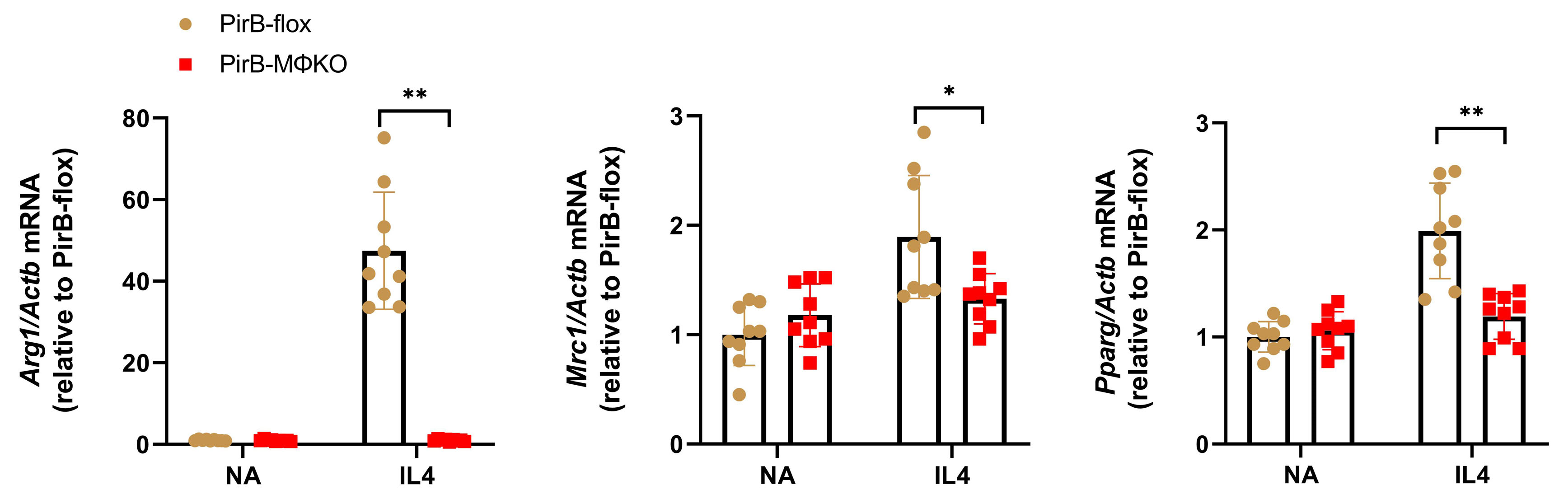


**Figure S12**


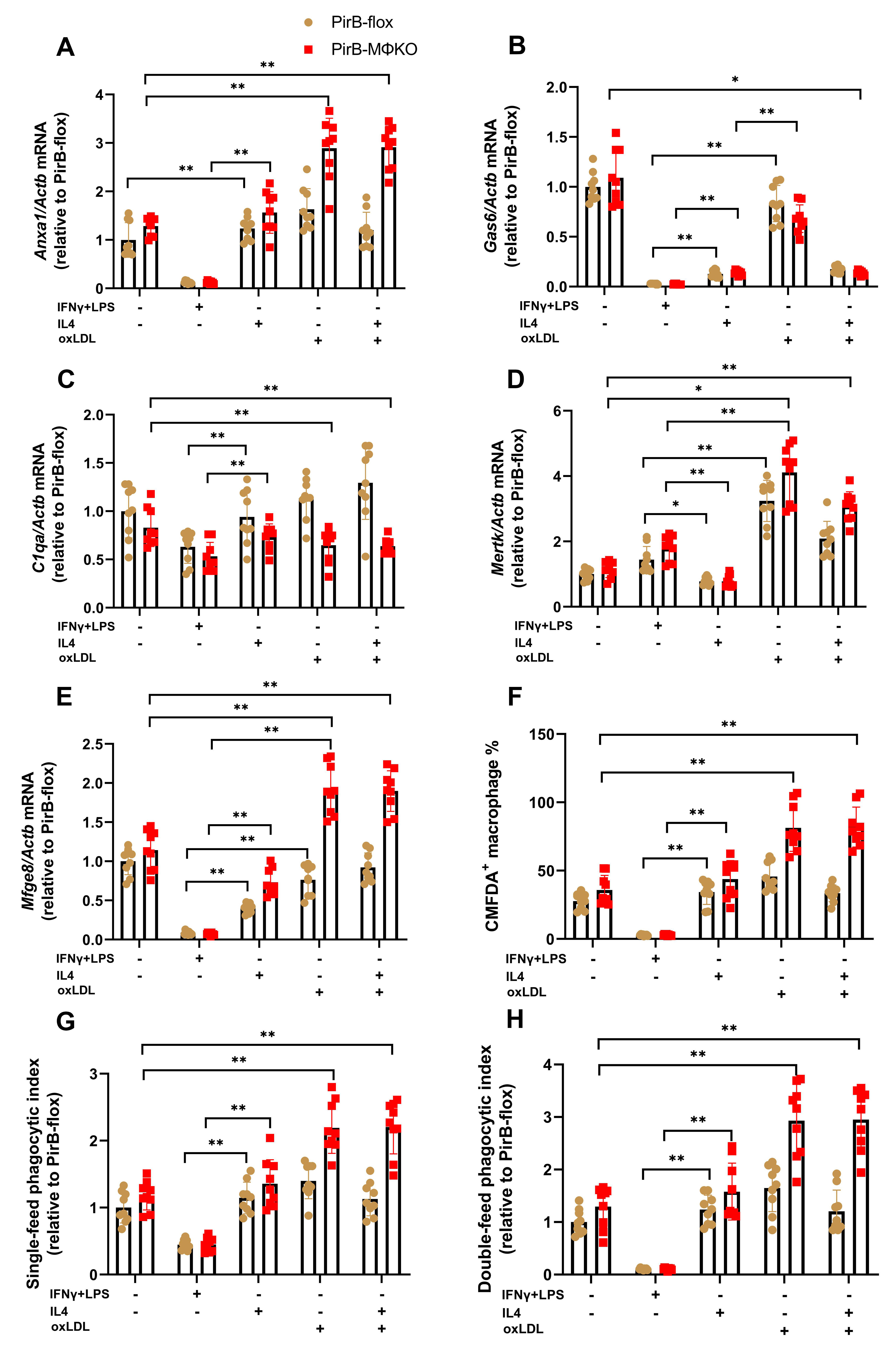


**REFERENCES FOR SUPPLEMENTARY INFORMATION**

Altermann, E., and Klaenhammer, T.R. (2005). PathwayVoyager: pathway mapping using the Kyoto Encyclopedia of Genes and Genomes (KEGG) database. *BMC genomics* 6**,** 60.

Bindea, G., Galon, J., and Mlecnik, B. (2013). CluePedia Cytoscape plugin: pathway insights using integrated experimental and in silico data. *Bioinformatics* 29**,** 661-663.

Bochner, D.N., Sapp, R.W., Adelson, J.D., Zhang, S., Lee, H., Djurisic, M., Syken, J., Dan, Y., and Shatz, C.J. (2014). Blocking PirB up-regulates spines and functional synapses to unlock visual cortical plasticity and facilitate recovery from amblyopia. *Science translational medicine* 6**,** 258ra140-258ra140.

Cui, S., Wang, C., Bai, W., Li, J., Pan, Y., Huang, X., Yang, H., Feng, Z., Xiang, Q., and Fei, L. (2020). CD1d1 intrinsic signaling in macrophages controls NLRP3 inflammasome expression during inflammation. *Science advances* 6**,** eaaz7290.

Doran, A.C., Ozcan, L., Cai, B., Zheng, Z., Fredman, G., Rymond, C.C., Dorweiler, B., Sluimer, J.C., Hsieh, J., and Kuriakose, G. (2017). CAMKIIγ suppresses an efferocytosis pathway in macrophages and promotes atherosclerotic plaque necrosis. *The Journal of clinical investigation* 127**,** 4075-4089.

Engchuan, W., Meechai, A., Tongsima, S., and Chan, J.H. (2016). Handling batch effects on cross-platform classification of microarray data. *International Journal of Advanced Intelligence Paradigms* 8**,** 59-76.

Franceschini, A., Szklarczyk, D., Frankild, S., Kuhn, M., Simonovic, M., Roth, A., Lin, J., Minguez, P., Bork, P., and Von Mering, C. (2012). STRING v9. 1: protein-protein interaction networks, with increased coverage and integration. *Nucleic acids research* 41**,** D808-D815.

Gough, P.J., Gomez, I.G., Wille, P.T., and Raines, E.W. (2006). Macrophage expression of active MMP-9 induces acute plaque disruption in apoE-deficient mice. *The Journal of clinical investigation* 116**,** 59-69.

Heo, K.-S., Cushman, H.J., Akaike, M., Woo, C.-H., Wang, X., Qiu, X., Fujiwara, K., and Abe, J.-I. (2014). ERK5 activation in macrophages promotes efferocytosis and inhibits atherosclerosis. *Circulation* 130**,** 180-191.

Higashi, Y., Sukhanov, S., Parthasarathy, S., and Delafontaine, P. (2008). The ubiquitin ligase Nedd4 mediates oxidized low-density lipoprotein-induced downregulation of insulin-like growth factor-1 receptor. *American Journal of Physiology-Heart and Circulatory Physiology* 295**,** H1684-H1689.

Johnson, W.E., Li, C., and Rabinovic, A. (2007). Adjusting batch effects in microarray expression data using empirical Bayes methods. *Biostatistics* 8**,** 118-127.

Kohl, M., Wiese, S., and Warscheid, B. (2011). "Cytoscape: software for visualization and analysis of biological networks," in *Data mining in proteomics*. Springer), 291-303.

Langfelder, P., and Horvath, S. (2008). WGCNA: an R package for weighted correlation network analysis. *BMC bioinformatics* 9**,** 559.

Lardenoye, J., Delsing, D., De Vries, M., Deckers, M., Princen, H., Havekes, L., Van Hinsbergh, V., Van Bockel, J., and Quax, P. (2000). Accelerated atherosclerosis by placement of a perivascular cuff and a cholesterol-rich diet in ApoE* 3Leiden transgenic mice. *Circulation research* 87**,** 248-253.

Lu, Y., Jiang, Z., Dai, H., Miao, R., Shu, J., Gu, H., Liu, X., Huang, Z., Yang, G., and Chen, A.F. (2018). Hepatic leukocyte immunoglobulin‐like receptor B4 (LILRB4) attenuates nonalcoholic fatty liver disease via SHP1‐TRAF6 pathway. *Hepatology* 67**,** 1303-1319.

Luo, W., and Brouwer, C. (2013). Pathview: an R/Bioconductor package for pathway-based data integration and visualization. *Bioinformatics* 29**,** 1830-1831.

Sukhanov, S., Higashi, Y., Shai, S.-Y., Itabe, H., Ono, K., Parthasarathy, S., and Delafontaine, P. (2006). Novel effect of oxidized low-density lipoprotein: cellular ATP depletion via downregulation of glyceraldehyde-3-phosphate dehydrogenase. *Circulation research* 99**,** 191-200.

Sukhanov, S., Higashi, Y., Shai, S.-Y., Vaughn, C., Mohler, J., Li, Y., Song, Y.-H., Titterington, J., and Delafontaine, P. (2007). IGF-1 reduces inflammatory responses, suppresses oxidative stress, and decreases atherosclerosis progression in ApoE-deficient mice. *Arteriosclerosis, thrombosis, and vascular biology* 27**,** 2684-2690.

Tacke, F., Alvarez, D., Kaplan, T.J., Jakubzick, C., Spanbroek, R., Llodra, J., Garin, A., Liu, J., Mack, M., and Van Rooijen, N. (2007). Monocyte subsets differentially employ CCR2, CCR5, and CX3CR1 to accumulate within atherosclerotic plaques. *The Journal of clinical investigation* 117**,** 185-194.

Tweedie, S., Ashburner, M., Falls, K., Leyland, P., Mcquilton, P., Marygold, S., Millburn, G., Osumi-Sutherland, D., Schroeder, A., and Seal, R. (2008). FlyBase: enhancing Drosophila gene ontology annotations. *Nucleic acids research* 37**,** D555-D559.

Wang, W.Z., Jones, A.W., Wang, M., Durante, W., and Korthuis, R.J. (2013). Preconditioning with soluble guanylate cyclase activation prevents postischemic inflammation and reduces nitrate tolerance in heme oxygenase-1 knockout mice. *American Journal of Physiology-Heart and Circulatory Physiology* 305**,** H521-H532.

Wang, Y., Gao, H., Loyd, C.M., Fu, W., Diaconu, D., Liu, S., Cooper, K.D., Mccormick, T.S., Simon, D.I., and Ward, N.L. (2012). Chronic skin-specific inflammation promotes vascular inflammation and thrombosis. *Journal of Investigative Dermatology* 132**,** 2067-2075.

Yurdagul Jr, A., Subramanian, M., Wang, X., Crown, S.B., Ilkayeva, O.R., Darville, L., Kolluru, G.K., Rymond, C.C., Gerlach, B.D., and Zheng, Z. (2020). Macrophage metabolism of apoptotic cell-derived arginine promotes continual efferocytosis and resolution of injury. *Cell metabolism* 31**,** 518-533. e510.

Zhang, X., Goncalves, R., and Mosser, D.M. (2008). The isolation and characterization of murine macrophages. *Current protocols in immunology* 83**,** 14.11. 11-14.11. 14.
